# Dynamic competition between SARS-CoV-2 NSP1 and mRNA on the human ribosome inhibits translation initiation

**DOI:** 10.1073/pnas.2017715118

**Published:** 2021-01-21

**Authors:** Christopher P. Lapointe, Rosslyn Grosely, Alex G. Johnson, Jinfan Wang, Israel S. Fernández, Joseph D. Puglisi

**Affiliations:** ^a^Department of Structural Biology, Stanford University School of Medicine, Stanford, CA 94305;; ^b^Department of Chemical and Systems Biology, Stanford University School of Medicine, Stanford, CA 94305;; ^c^Department of Biochemistry and Molecular Biophysics, Columbia University, New York City, NY 10032

**Keywords:** SARS-CoV-2, NSP1, human ribosome, eukaryotic translation initiation, single-molecule fluorescence

## Abstract

Severe acute respiratory syndrome coronavirus 2 (SARS-CoV-2) is a beta-CoV that recently emerged as a human pathogen and is the causative agent of the COVID-19 pandemic. A molecular framework of how the virus manipulates host cellular machinery to facilitate infection remains unclear. Here, we focus on SARS-CoV-2 NSP1, which is proposed to be a virulence factor that inhibits protein synthesis by directly binding the human ribosome. We demonstrate biochemically that NSP1 inhibits translation of model human and SARS-CoV-2 messenger RNAs (mRNAs). NSP1 specifically binds to the small (40S) ribosomal subunit, which is required for translation inhibition. Using single-molecule fluorescence assays to monitor NSP1–40S subunit binding in real time, we determine that eukaryotic translation initiation factors (eIFs) allosterically modulate the interaction of NSP1 with ribosomal preinitiation complexes in the absence of mRNA. We further elucidate that NSP1 competes with RNA segments downstream of the start codon to bind the 40S subunit and that the protein is unable to associate rapidly with 80S ribosomes assembled on an mRNA. Collectively, our findings support a model where NSP1 proteins from viruses in at least two subgenera of beta-CoVs associate with the open head conformation of the 40S subunit to inhibit an early step of translation, by preventing accommodation of mRNA within the entry channel.

## Significance

SARS-CoV-2 is the causative agent of the COVID-19 pandemic. A molecular framework for how the virus manipulates host cellular machinery to facilitate infection is needed. Here, we integrate biochemical and single-molecule strategies to reveal molecular insight into how NSP1 from SARS-CoV-2 inhibits translation initiation. NSP1 directly binds to the small (40S) subunit of the human ribosome, which is modulated by human initiation factors. Further, NSP1 and mRNA compete with each other to bind the ribosome. Our findings suggest that the presence of NSP1 on the small ribosomal subunit prevents proper accommodation of the mRNA. How this competition disrupts the many steps of translation initiation is an important target for future studies.

Beta-coronaviruses (CoVs) are a family of RNA viruses that include human pathogens (1). In the last two decades, two beta-CoVs have emerged from animal hosts to cause epidemic diseases of the human respiratory tract: severe acute respiratory syndrome (SARS-CoV, in 2002) (2, 3) and Middle East respiratory syndrome (MERS-CoV, in 2012) (4). A third beta-CoV emerged in late 2019—SARS-CoV-2—that is responsible for the ongoing COVID-19 pandemic (5). Given the lack of effective therapies against SARS-CoV-2, there is an urgent need for a molecular understanding of how the virus manipulates the machineries present in human cells.

SARS-CoV-2 and the closely related SARS-CoV have single-stranded, positive-sense RNA genomes nearly 30 kb in length (6, 7). Upon entry of a virion into human cells, the genomic RNA is released into the cytoplasm where it must hijack human translation machinery to synthesize viral proteins (8). As the genomic RNA has a 7-methylguanosine (m^7^G) cap on the 5′ terminus, viral protein synthesis likely proceeds via a process reminiscent of that which occurs on typical human messenger RNAs (mRNAs) (9). However, as viral proteins accumulate, human translation is inhibited and host mRNAs are destabilized, which facilitates suppression of the host immune response (10–13).

Studies on SARS-CoV have implicated nonstructural protein 1 (NSP1), the first encoded viral protein, as a virulence factor with a key role in the shutdown of host translation (10, 11, 14). In infected cells or upon its ectopic expression, NSP1 inhibits human translation, which is dependent on its association with the small (40S) subunit of the human ribosome (12–17). In a linked but separable activity, NSP1 destabilizes at least a subset of human mRNAs, likely via recruitment of an unidentified human endonuclease (12, 13, 15, 16, 18). NSP1 from SARS-CoV-2 is expected to employ similar mechanisms, given its ∼85% sequence identity with the SARS-CoV protein. Indeed, SARS-CoV-2 NSP1 inhibits translation by binding to the 40S subunit (19–21). Thus, NSP1 has a near-singular ability to disrupt host gene expression dramatically; yet, the mechanism by which this inhibition occurs is not clear.

The 40S subunit is the nexus of translation initiation, recruiting an m^7^G-capped mRNA through a multistep, eukaryotic initiation factor (eIF)-mediated process. Prior to recruitment of an mRNA, the 40S subunit is bound by numerous eIFs, which include eIF1, eIF1A, eIF3, eIF5, and the ternary complex (TC) of eIF2–GTP–methionine initiator transfer RNA (tRNA_i_^Met^) (22). The eIFs make extensive contacts with the 40S subunit, including the ribosomal A and P sites (23, 24). They also manipulate the dynamics of the 40S head region to facilitate mRNA recruitment, which has structural consequences at both the mRNA entry (3′ side of mRNA) and exit (5′ end of mRNA) channels. Following mRNA recruitment and directional scanning of the 5′ untranslated region (UTR) to a start codon, a series of compositional and conformational changes occur (25, 26). This ultimately repositions the 40S subunit head into the closed conformation and accommodates the anticodon stem loop of the initiator tRNA at the start codon (23–26), enabling recruitment of the 60S subunit and entry into the elongation phase.

Recently, structures of NSP1 bound to human ribosomes have been reported (19–21), including 40S preinitiation and 80S ribosomal complexes. In all instances, the N-terminal globular domain of NSP1 is flexibly localized to the solvent-exposed surface of the 40S subunit, near the entrance to the mRNA entry channel (*SI Appendix*, Fig. S1*A*). This domain is anchored by the two most C-terminal α-helices of NSP1, which were dynamic and unstructured in the free SARS-CoV NSP1 structure solved by NMR (27); in the NSP1–40S subunit complex, these helices were well resolved and docked within the mRNA entry channel, where they contact ribosomal proteins uS3 and uS5, and helix 18 of the 18S ribosomal RNA (rRNA). As noted above, this location on the ribosome is structurally flexible, adopting open and closed states upon swiveling of the 40S subunit head (23–25). The position of NSP1 in the mRNA channel may also conflict with the position of fully accommodated mRNA. Thus, the intrinsic dynamics of translation initiation present many opportunities and obstacles for NSP1 association with the ribosome, and its subsequent inhibition of translation.

Here, we merge biochemical and single-molecule approaches to probe the molecular function of SARS-CoV-2 NSP1 and its interaction with the human ribosome. We showed that NSP1 potently inhibited translation of human and SARS-CoV-2 model mRNAs, and determined how the NSP1–40S subunit interaction was modulated by eIFs and mRNA. Our results reveal allosteric control of NSP1 association by key eIFs and identify a conformation of the ribosomal subunit compatible with rapid NSP1 association. They also define the dynamic competition between NSP1 and mRNA to bind the ribosome. When synthesized with recent structures, our study suggests a mechanism for how NSP1 inhibits translation initiation.

## Results

### NSP1 Inhibited Translation of Host and SARS-CoV-2 Model mRNAs.

**Fig. 1.**
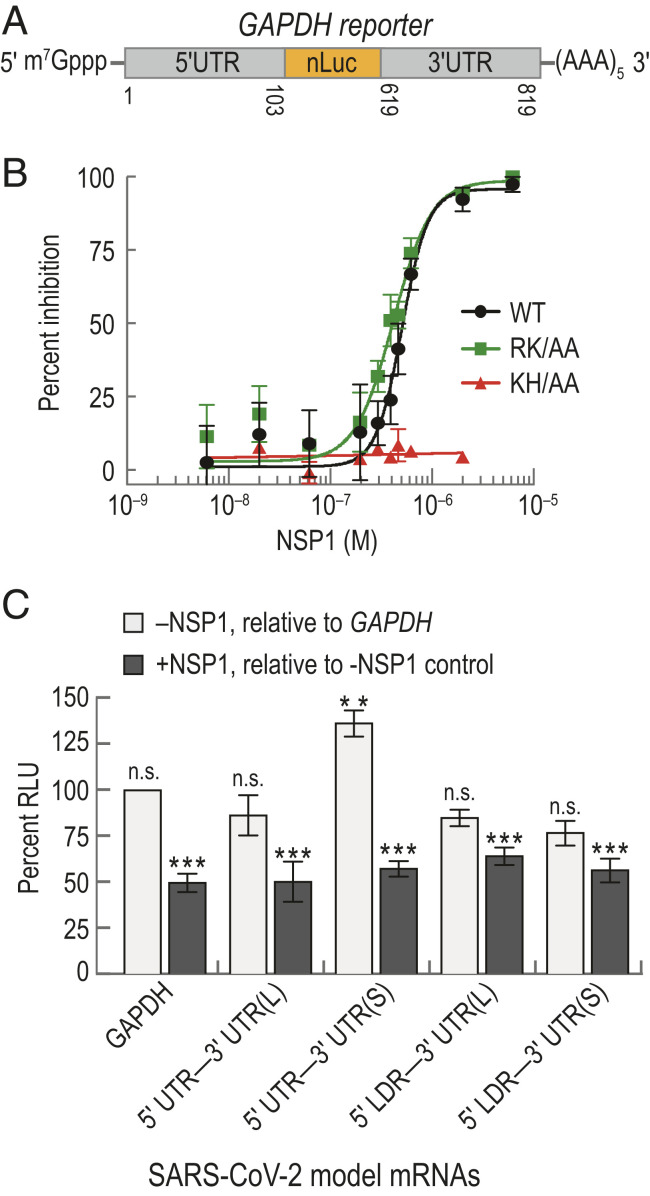
NSP1 from SARS-CoV-2 inhibited translation. (*A*) Schematic of the host model mRNA used in HeLa cell-free IVT assays. The nLuc coding sequence of the mRNA was flanked by the 5′ and 3′ UTRs of human GAPDH. Numbers refer to the nucleotide position in NCBI GenBank accession: AF261085. (*B*) NSP1 dose–response analysis of GAPDH reporter mRNA IVT in HeLa extract treated with either wild-type (WT; *n* = 3), a predicted ribosome-binding−deficient mutant (KH/AA; *n* = 2), or an RNA cleavage-deficient mutant (RK/AA; *n* = 2) NSP1. The mean response ± SEM (symbols, error bars) and curve fits (lines) from nonlinear regression analysis of the data are plotted. WT IC_50_ = 510 ± 20 nM (95% CI; *R*^2^ = 0. 83), and RK/AA IC_50_ = 420 ± 11 nM (95% CI; *R*^2^ = 0.89). (*C*) Plot of the mean nLuc relative light unit (RLU) signal from cell-free translation of host and viral reporter mRNAs in the absence and presence (400 nM) of wild-type NSP1. Without NSP1 (light gray), mean translational activities (percent RLU) were compared to GAPDH reporter mRNA in the absence of NSP1 (** = *P* ≤ 0.0006; and n.s. = *P* ≥ 0.2, one-way ANOVA). GAPDH (*n* = 6), 5′ UTR−3′ UTR(S) mRNA (*n* = 3), 5′ UTR−3′ UTR(L) (*n* = 3), and 5′ LDR reporter mRNAs (*n* = 5). In the presence of NSP1 (dark gray), samples were compared to a control reaction that lacked NSP1 (∗∗∗≤p≤0.0008, *t* test). GAPDH (*n* = 6), viral 5′ UTR mRNAs (*n* = 3), and viral 5′ LDR mRNAs (*n* = 2). Error bars represent SEM.

We first recapitulated and then quantified the extent of translation inhibition achieved by NSP1 from SARS-CoV-2. Our approach was to employ a cell-free in vitro translation (IVT) assay using HeLa cellular extract and purified protein (*SI Appendix*, Fig. S1*B*). As a model of a host mRNA, the 5′ and 3′ UTRs from human glyceraldehyde-3-phosphate dehydrogenase (GAPDH) mRNA were fused to a nanoluciferase (nLuc) open-reading frame (ORF) (Fig. 1*A*). The extract displayed m^7^G cap dependent enhancement of translation, a key feature of protein synthesis in cells (*SI Appendix*, Fig. S1*C*). When NSP1 was added to the extract, we observed a concentration-dependent reduction in nLuc activity, with a half-maximal inhibitory concentration (IC_50_) of 510 ± 20 nM (95% CI; *R*^2^ = 0. 83) (Fig. 1*B*). As predicted based on the SARS-CoV protein (16), substitution of NSP1 residues implicated in potential mRNA destabilization yielded translation inhibition similar to the wild-type protein (RK124-125AA; IC_50_ ≈ 420 ± 11 nM, 95% CI, *R*^2^ = 0.89), whereas substitution of residues implicated in ribosome binding alleviated the inhibition (KH164-165AA; Fig. 1*B* and *SI Appendix*, Fig. S1*D*). The inhibitory effect of NSP1 on protein synthesis therefore likely is meditated by a high-affinity, specific NSP1−ribosome interaction, independent of mRNA degradation.

In the context of infection, full-length SARS-CoV-2 genomic RNA and its subgenomic mRNAs must be translated in the presence of NSP1 protein. To examine whether RNA elements within the viral UTRs facilitate evasion of NSP1-mediated inhibition, we constructed model SARS-CoV-2 mRNAs in which the nLuc ORF was flanked on the 5′ end by either the full-length viral 5′ UTR or the subgenomic 5′ leader sequence (LDR) (*SI Appendix*, Fig. S1*E*). At the 3′ end, we fused two different versions of the 3′ UTR, beginning after the stop codon for N protein (L) or ORF10 (S), to account for ambiguity in ORF10 coding potential (28). Translation of all four model viral mRNAs was reduced significantly by ∼50% upon addition of 400 nM NSP1 relative to reactions that lacked NSP1 (*P* ≤ 0.0008, unpaired *t* test) (Fig. 1*C*). This level of inhibition was similar to the inhibition observed for the GAPDH reporter mRNA (*P* ≥ 0.2, one-way ANOVA), consistent with the IC_50_ determination above. However, translation of the 5′ UTR−3′ UTR(S) model viral mRNA was modestly increased (∼36%) relative to the host and other viral reporters in our extract-based assays (*P* ≤ 0.0006, one-way ANOVA) (Fig. 1*C*). This may suggest that enhanced translational activity of viral RNAs relative to host mRNAs could play a role in infection and evasion of NSP1 action. Regardless, NSP1 is a potent inhibitor of translation.

### NSP1 Stably Associated with Ribosomal Preinitiation Complexes.

To examine the interaction between NSP1 and the human 40S ribosomal subunit, we first employed native gel shift assays using purified NSP1, ribosomes, and eIFs. Using an 11-amino acid ybbR tag, single cyanine dye fluorophores were conjugated site specifically onto NSP1 (*SI Appendix*, Fig. S2 *A* and *B*) (29, 30). When incubated with increasing concentrations of ribosomal subunits, the amount of fluorescently labeled NSP1 that comigrated with 40S subunits increased (*SI Appendix*, Fig. S2*C*). In contrast, NSP1 did not comigrate with human 60S or yeast 40S subunits (*SI Appendix*, Fig. S2*D*). To probe specificity further, we performed competition assays: NSP1(KH/AA) was unable to block the NSP1–40S subunit interaction, whereas either wild-type NSP1 or NSP1(RK/AA) at 150-fold molar excess prevented comigration of labeled NSP1 with human 40S subunits (*SI Appendix*, Fig. S2*E*). Encouraged, we probed how NSP1 binding to the 40S subunit was affected by the presence of 6 µM eIF1 and/or eIF1A, since both have been visualized in structures of NSP1–40S subunit complexes (19, 20). eIF3j also was selected, as it binds the 40S subunit with high affinity near the mRNA entry channel (31–33). Inclusion of eIF1 increased the intensity of the NSP1–40S subunit band approximately twofold (mean ≈ 2 ± 0.4, 95% CI), while eIF3j eliminated the band (*SI Appendix*, Fig. S2 *F*–*H* and Table S1). eIF1A had little impact on formation of the NSP1–40S subunit complex (*SI Appendix*, Fig. S2 *F* and *I*). NSP1 therefore specifically interacts with the human 40S subunit, which is modulated inversely by two key eIFs, perhaps through induced changes in ribosome conformation (23, 24).

**Fig. 2.**
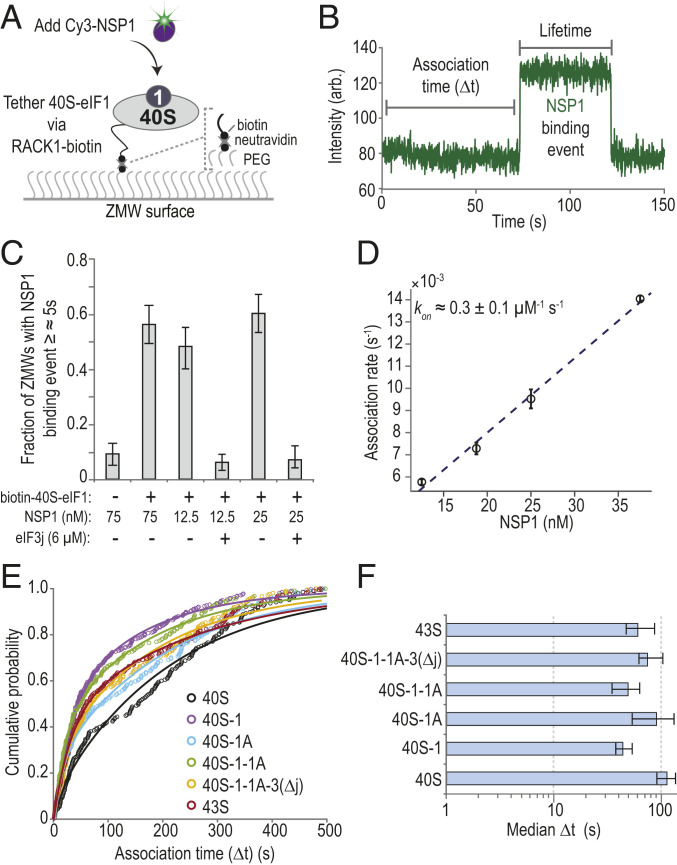
NSP1 associated with 40S subunits and most ribosomal preinitiation complexes. (*A*) Experimental setup. Using a ZMW system, 40S ribosomal subunits biotinylated on RACK1 were tethered to a neutravidin-coated imaging surface within thousands of individual ZMWs (also see *SI Appendix*, Fig. S3*B*). Upon start of data acquisition, Cy3-NSP1 (N-terminal ybbR tag) was added, and fluorescence intensities were monitored. (*B*) Example single-molecule fluorescence trace that depicts association of Cy3-NSP1 with a tethered eIF1–40S subunit complex. Prior to tethering, 40S subunits were incubated with 30-fold molar excess eIF1. During imaging, eIF1 was present at 1 µM. The association time (Δ*t*) was defined as the time elapsed from the addition of Cy3-NSP1 until the burst of Cy3 fluorescence (green), which signified NSP1 association. The lifetime was defined as the duration of the Cy3 fluorescence signal. (*C*) Plot of the fraction of ZMWs that contained at least one Cy3-NSP1 binding event ≥∼5 s in duration in the indicated conditions at 20 °C. Error bars represent 99% CI. (*D*) Plot of apparent association rates (open circles) of Cy3-NSP1 with tethered eIF1–40S subunit complexes at the indicated NSP1 concentrations at 20 °C. The dashed line represents a fit from linear regression analysis (adjusted *R*^2^ = 0.99), with a slope of 0.3 ± 0.1 and *y* intercept of 0.0013 ± 0.002 (errors represent 95% CI). Error bars on the open circles represent 95% CI of the rates. (*E*) Plot of the cumulative probability of Cy3-NSP1 association times with the indicated ribosomal preinitiation complexes. Cy3-NSP1 was present at 25 nM (final concentration), and the temperature was 30 °C. The eIFs were preincubated with 40S subunits, and they were included at molar excess relative to 40S subunits during tethering and imaging to promote formation of the indicated complexes. The proteins eIF1, eIF1A, and eIF5 were present at 1 µM; the eIF2-GMPPNP-Met-tRNA_i_^Met^ ternary complex at 100 nM; and eIF3Δj at 50 nM. Lines represent fits to double-exponential functions. See *SI Appendix*, Table S2 for samples sizes and the parameters for relevant fits. (*F*) Plot of Cy3-NSP1 median association times (light blue) with the indicated ribosomal preinitiation complexes. Error bars represent 95% CI of the median values.

To define the kinetics of NSP1 binding to 40S subunits and how they are affected by eIFs, we established a single-molecule assay to monitor NSP1 association with ribosomal preinitiation complexes directly in real time. First, biotin was attached to purified 40S subunits that contained the ybbR tag on the ribosomal protein RACK1 (*SI Appendix*, Fig. S3*A*) (34). We then tethered preassembled eIF1–40S(biotin) subunit complexes to thousands of zero-mode waveguide (ZMW) surfaces coated with neutravidin (*SI Appendix*, Fig. S3*B*) (35). Upon the start of data acquisition, Cy3-NSP1 was added, which inhibited translation similar to the wild-type NSP1 (*SI Appendix*, Fig. S3 *C* and *D*). Association of the protein with the 40S subunit was manifested by a burst of Cy3 fluorescence (Fig. 2 *A* and *B*). When NSP1 was delivered to tethered complexes at 75 nM, the majority of ZMWs (56 ± 7%) contained at least one NSP1 binding event (≥∼5 s in length) (Fig. 2*C* and *SI Appendix*, Table S2). This signal was specific, as the number of ZMWs with binding events was reduced in the absence of the tethered complex (9 ± 4%). Similarly, preincubation with 2.5 µM eIF3j reduced NSP1 binding at two different concentrations to baseline levels (from 48 ± 7% and 60 ± 7% to 6 ± 3% and 7 ± 3%). Results consistent with specific binding also were obtained using total internal reflection fluorescence microscopy (TIRFM) at NSP1–40S subunit equilibrium (*SI Appendix*, Fig. S3 *E* and *F*). Thus, our assay directly monitored real-time association of NSP1 with tethered 40S ribosomal complexes and further demonstrated competition by eIF3j for NSP1–40S subunit complex formation.

NSP1 bound the eIF1–40S subunit complex with high affinity. As predicted for a simple bimolecular interaction, NSP1 association times (Δt, the time elapsed from its addition until appearance of Cy3 signal) decreased with increasing concentration of NSP1 at 20 °C (*SI Appendix*, Fig. S3*G*). Linear regression analysis of the observed rates at various NSP1 concentrations yielded a bimolecular association rate of 0.3 ± 0.1 µM^−1^⋅s^−1^ (Fig. 2*D* and *SI Appendix*, Table S2). The observed lifetime of the NSP1–40S subunit interaction (the duration of the Cy3 signal) was dependent on the power of the excitation laser (*SI Appendix*, Fig. S3 *H* and *I*), which indicated that our measurements may be limited by dye photostability. Nevertheless, with the slowest rate of dissociation we measured as a lower bound $\left(k_{off}\approx 0.0042\pm 0.001\;s^{-1}\right)$, we estimated that the equilibrium dissociation constant (*K*_*D*_) of the NSP1 interaction with eIF1–40S subunit complexes was ≤∼10 nM at 20 °C, similar to that of eIFs (36).

NSP1 rapidly and stably associated with various ribosomal preinitiation complexes. Given the threshold-like temperature dependence of NSP1 association with the eIF1–40S subunit complex (*SI Appendix*, Fig. S3*J*), we measured NSP1 association times and lifetimes with 40S subunits in complex with canonical eIFs at 30 °C. Consistent with our gel-based assays, the median NSP1 association time (Δt) at 25 nM decreased about twofold in the presence of eIF1 relative to 40S subunits alone (38 s to 54 s versus 91 s to 137 s; see *Materials and Methods (Condensed)*) (Fig. 2 *E* and *F*). Further inclusion of eIF1A, eIF3 that lacked the 3j subunit (eIF3Δj), eIF5, and/or an eIF2–tRNA_i_^Met^–GMPPNP ternary complex (TC-GMPPNP) also yielded modest reductions in NSP1 Δt. NSP1 lifetimes on the various eIF–40S subunit complexes were similar and likely limited by dye photostability in the imaging conditions (*SI Appendix*, Fig. S3*K*). The eIF-mediated modulation of NSP1 association rates with the 40S subunit—particularly by eIF1 as it binds at the ribosomal P site distally to the NSP1 binding site—suggested that NSP1 may associate with a particular conformation of the 40S ribosomal subunit (*SI Appendix*, Fig. S3*L*).

### NSP1 Preferentially Associated with the Open Head Conformation of the 40S Subunit.

**Fig. 3.**
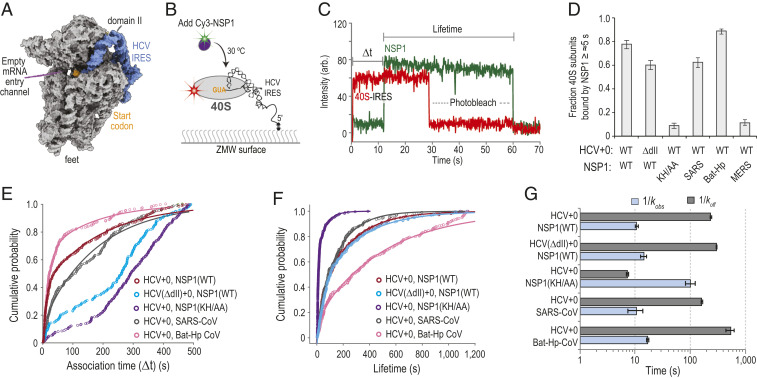
NSP1 preferentially associated with the open head conformation of the 40S subunit. (*A*) Model of the human 40S subunit (gray) bound by the HCV IRES (blue) (PDB ID code 5A2Q) (38). This model of the IRES ends at the start codon (AUG, highlighted in orange), leaving the mRNA entry channel of the 40S subunit empty. Domain II of the IRES holds the head of the 40S subunit in the open conformation. (*B*) Schematic of the single-molecule fluorescence assay. The 40S ribosomal subunits were labeled with Cy5 dye via RACK1-ybbR. Preformed IRES–40S-Cy5 complexes were tethered to the ZMW imaging surface. At the start of data acquisition, Cy3-NSP1 (N-terminal ybbR tag) was added at 25 nM (final concentration) at 30 °C. (*C*) Example single-molecule fluorescence trace that depicts a tethered 40S–HCV+0 complex and subsequent association of NSP1. The 40S subunit and ybbR-NSP1 were labeled with Cy5 (red) and Cy3 (green) dyes, respectively. Loss of fluorescence signal due to dye photobleaching is indicated. Raw fluorescence intensities were corrected in this image to set baseline intensities to zero for presentation. The association time (Δ*t*) was defined as time elapsed from the addition of Cy3-NSP1 until the burst of Cy3 fluorescence (green), which signified NSP1 association. The lifetime was defined as the duration of the Cy3 fluorescence signal. (*D*) Plot of the fraction of the indicated IRES–40S subunit complexes bound at least once by the indicated NSP1 protein for ≥∼5 s. Error bars represent 99% CI. WT, SARS-CoV-2 NSP1; KH/AA, SARS-CoV-2 NSP1(KH/AA). (*E* and *F*) Plot of the cumulative probability of observed Cy3-NSP1 association times (*E*) and lifetimes (*F*) with the indicated IRES–40S subunit complexes at 30 °C. The indicated Cy3-NSP1 was added at 25 nM (final concentration) in all experiments. Lines represent fits to double-exponential functions. See *SI Appendix*, Table S3 for samples sizes and the parameters for relevant fits. Association times were determined with the excitation laser (532 nm) at 0.6 µW/µm^2^, whereas lifetimes were determined at the further reduced power of 0.1 µW/µm^2^ to enhance dye stability. (*G*) Plot of the reciprocal apparent association (*k*_*obs*_) (light blue) and dissociation (*k*_*off*_) (light gray) rates of the indicated NSP1 binding to the indicated IRES–40S subunit complexes. Rates were derived from fits of data to double-exponential functions, with the fast association rate and predominate lifetime reported here. See *SI Appendix*, Table S3 for samples sizes and all parameters from relevant fits. Error bars represent 95% CI.

To examine whether the conformation of the mRNA entry channel impacted NSP1 association, we leveraged the internal ribosome entry site (IRES) from hepatitis C virus (HCV). This structured RNA directly binds to the human 40S subunit with high affinity (2 nM to 4 nM) (37). It also contains a flexible segment (domain II) distal to the NSP1 binding site that is dispensable for affinity but swivels the head of the ribosomal subunit to open the entry channel (38–40) (Fig. 3*A* and *SI Appendix*, Fig. S4 *A* and *B*). We generated HCV IRES RNAs with and without domain II (ΔdII) that were 5′ biotinylated and contained zero nt downstream (3′) of the start codon (HCV+0), which left the entry channel free of mRNA. Following incubation of HCV+0 or HCV(ΔdII)+0 RNAs with fluorescently labeled (Cy5 dye) ribosomal subunits, 25 nM of Cy3-NSP1 was delivered to IRES–40S subunit complexes tethered in ZMWs at 30 °C (Fig. 3 *B* and *C*). NSP1 efficiently (77 ± 3%), rapidly $\left(k_{obs}\approx 0.095\pm 0.006\;s^{-1}\right)$, and stably $\left(k_{off}\leq 0.0043\pm 0.0001\;s^{-1}\right)$ associated with the 40S–HCV+0 complex (Fig. 3 *D*–*G* and *SI Appendix*, Table S3), with an estimated *K*_*D*_ of ≤∼1 nM. Notably, this association rate was approximately threefold faster than with the eIF1–40S complex $\left(k_{obs}\approx 0.035\pm 0.002\;s^{-1}\right)$ (*SI Appendix*, Fig. S4*C*), the next fastest rate we have observed. Despite a similar association efficiency (60 ± 4%), there was a striking delay in NSP1 association with the 40S–HCV(ΔdII)+0 complex with multiphasic behavior (median Δ*t* ≈ 242 s to 277 s) (Fig. 3 *D* and *E* and *SI Appendix*, Fig. S4*D*). Further analysis indicated that only a small population of complexes (∼10%) were competent for rapid NSP1 association $\left(k_{obs}\approx 0.07\pm 0.01\;s^{-1};\;k_{off}\leq 0.003\pm 0.0001\right)$ (*SI Appendix*, Fig. S4*E*). Thus, the known shift in conformational equilibrium of the entry channel from the open to closed state in the IRES(ΔdII)–40S subunit complex is incompatible with rapid NSP1 association.

We also probed whether NSP1 from closely related (SARS-CoV) and more divergent beta-CoVs (Bat-Hp-CoV and MERS-CoV) rapidly associate with the ribosome, despite changes in the composition and length of the C-terminal tail (*SI Appendix*, Fig. S1*D*). As expected given its conservation and translation−inhibition activity (*SI Appendix*, Fig. S4*F*), NSP1 from SARS-CoV bound the 40S–HCV+0 complex with similar efficiency (62 ± 4%) and kinetics (*k_obs_* ≈ 0.096 ± 0.04 s^−1^; *k_off_* ≤ 0.006 ± 0.0002 s^−1^; *K_D_* ≤ ∼ 2 nM) as the SARS-CoV-2 protein when added at 25 nM (Fig. 3 *D*–*G* and *SI Appendix*, Fig. S4*G* and Table S3). Intriguingly, similar results were obtained with the more divergent Bat-Hp-CoV NSP1 89 ± 2%; *k_obs_* ≈ 0.060 ± 0.003 s^−1^; *k_off_* ≤0.002 ± 0.0004 s^−1^; KD ≤ ∼ 1 nM) (Fig. 3 *D*–*G* and *SI Appendix*, Fig. S4*H*). Thus, the substitutions and three amino acid deletion in its C-terminal tail permit stable association with the human ribosome. In contrast, disruption of the conserved KH164-165 residues in SARS-CoV-2 NSP1 reduced its association with the 40S–HCV+0 subunit complex (9 ± 2%) to levels observed with the MERS-CoV protein (11 ± 2%) (Fig. 3*D* and *SI Appendix*, Fig. S4*I*), which lacks ribosome binding activity (41). The infrequent NSP1(KH/AA) binding events we did observe were slow to occur $\left(k_{obs}\approx 0.01\pm 0.003\;s^{-1}\right)$ and much shorter in duration $\left(k_{off}\approx 0.14\pm 0.008\;s^{-1}\right)$ relative to the wild-type protein (Fig. 3 *E*–*G* and *SI Appendix*, Fig. S4*J*). These findings indicate an at least 350-fold decrease in affinity for the mutant protein $\left(K_{D}\geq 350\;\mathrm{nM}\right)$, consistent with our native gel assays. Together, our data support a model where NSP1 proteins from the *Sarbecovirus* (SARS-like viruses) and *Hibecovirus* (Bat-Hp-CoV) subgenera of beta-CoVs (42) preferentially associate with the open head conformation of the 40S subunit to inhibit translation.

### mRNA within the Entry Channel of the 40S Subunit Inhibited NSP1 Association.

**Fig. 4.**
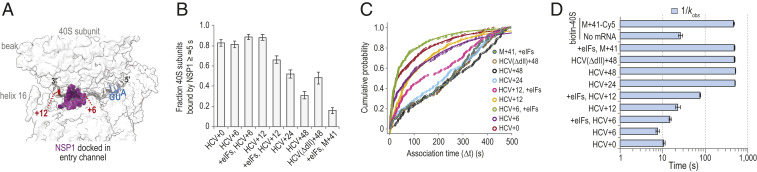
The mRNA within the entry channel of the 40S subunit inhibited SARS-CoV-2 NSP1 association. (*A*) Image of the intersubunit interface of the human 40S ribosomal subunit, with the approximate positions of mRNA (gray) and NSP1 (purple) modeled to show the predicted steric clash when segments of RNA longer than 6 nt are downstream of the start codon (blue). Models were aligned using ChimeraX (mmaker command) and PDB ID codes 6ZLW (19) and 6YAL (76). (*B*) Plot of the fraction of the indicated mRNA–40S subunit complexes bound at least once by SARS-CoV-2 NSP1 for ≥∼5 s. Error bars represent 99% CI. (*C*) Plot of the cumulative probability of observed Cy3-NSP1 association times with the indicated mRNA–40S indicates eIF1, eIF1A, eIF3Δj, eIF5, and TC(GMPPNP) were included at all stages of the experiment. Lines represent fits to single- or double-exponential functions. See *SI Appendix*, Table S4 for samples sizes and the parameters for relevant fits. (*D*) Plot of the reciprocal apparent association rates (*k*_*obs*_) (light blue) of SARS-CoV-2 NSP1 binding to the indicated mRNA–40S subunit complexes, derived from fits of the data to double-exponential functions, with the fast association rate reported here. See *SI Appendix*, Table S4 for samples sizes and the parameters from relevant fits. Error bars represent 95% CI.

Structural modeling suggested that mRNAs with more than 6 nt downstream (3′) of the start codon may occlude the NSP1 binding site in the entry channel (Fig. 4*A*). To examine this hypothesis, we generated additional versions of the HCV IRES with 6, 12, 24, and 48 nt after its start codon (*SI Appendix*, Fig. S5*A*). With 6 nt present (HCV+6), NSP1 efficiently (81 ± 3%) and rapidly associated $\left(k_{obs}\approx 0.13\pm 0.01\;s^{-1}\right)$ with tethered IRES–40S subunit complexes when added at 25 nM, nearly identical to the HCV+0 control $\left(82\pm 3\%;\;k_{obs}\approx 0.094\pm 0.006\;s^{-1}\right)$ (Fig. 4 *B*–*D* and *SI Appendix*, Table S4). In contrast, NSP1 associated with 40S–HCV+48 complexes less efficiently (31 ± 4%) and much more slowly $\left(k_{obs}\leq ∼0.002\;s^{-1}\right)$, which was followed by a more rapid departure from the complex $\left(k_{off}\approx 0.25\pm 0.02\;s^{-1}\right)$ (Fig. 4 *B*–*D* and *SI Appendix*, Fig. S5*B*). Similar inhibited, multiphasic association dynamics were observed with HCV+24 and HCV(ΔdII)+48. We reasoned that the relative lack of inhibition we observed on HCV+12 $\left(k_{obs}\approx 0.044\pm 0.007\;s^{-1}\right)$ was due to inefficient accommodation of the mRNA into the entry channel. Inclusion of eIFs used for initiation by the HCV IRES (eIF1, eIF1A, eIF5, eIF3Δj, and TC-GMPPNP) on HCV+12 further slowed, by eightfold, the NSP1 apparent association rate relative to HCV+0 $\left(k_{obs}\approx 0.013\pm 0.0004\;s^{-1}\right)$ (Fig. 4 *C* and *D*). Consequently, the *K*_*D*_ of the NSP1 interaction with the IRES–40S subunit complex was increased at least 2,000-fold (*K*_*D*_ ≥ 2 μM to 3 μM) by long segments of RNA downstream of the start codon.

To determine whether our findings were generalizable to other mRNAs, we performed analogous experiments in two formats using an unstructured model mRNA (M+41) that contained 41 nt downstream of the start codon (*SI Appendix*, Fig. S5*A*). In the first, 3′-biotinylated M+41 RNA bound to 40S-Cy5 subunits were tethered to the imaging surface (*SI Appendix*, Fig. S5 *C* and *D*). In the second, 40S-biotin subunits bound to fluorescently labeled M+41 were tethered (*SI Appendix*, Fig. S5 *E* and *F*). In both scenarios, we observed inefficient association of NSP1 with the mRNA–40S subunit complexes (16 ± 3% with biotin-M+41) and at least 48-fold increases in NSP1 association times ($k_{obs}\leq ∼0.002\;s^{-1}$ for both) relative to HCV+0 (Fig. 4 *B*–*D* and *SI Appendix*, Fig. S5 *G* and *H* and Table S4). NSP1 association dynamics again were multiphasic, which is behavior characteristic of strong inhibition by the model mRNA on NSP1 binding in these assays.

### A Förster Resonance Energy Transfer Signal Revealed Poor NSP1 Association with 80S Ribosomes Assembled on the Cricket Paralysis Virus IRES.

**Fig. 5.**
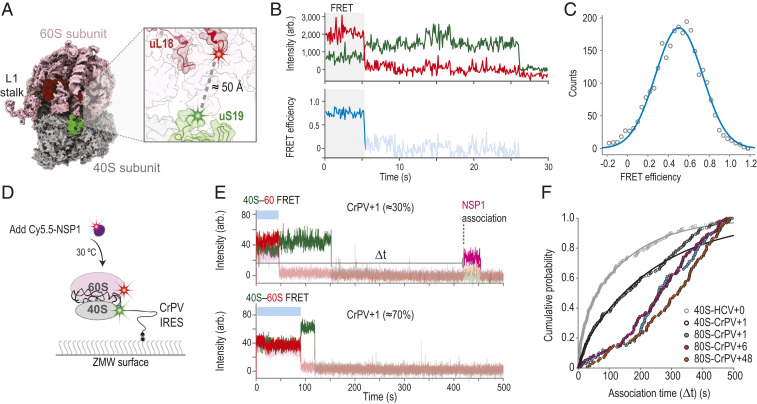
NSP1 inefficiently associated with 80S ribosomes assembled on the CrPV IRES. (*A*) Model of the human 80S ribosome (PDB ID code 4UG0). The 40S and 60S subunits are in gray and pink, respectively. The ybbR tag was fused to either the N terminus of uS19 (green, 40S) or the C terminus of uL18 (red, 60S). Based on available structural models, the ybbR tags were predicted to be within FRET distance in translation-competent 80S ribosomes. (*B*) Example fluorescent trace and calculated FRET efficiency plot. The 40S-ybbR-Cy3 and 60S-ybbR-Cy5 subunits were incubated with the CrPV IRES to assemble 80S ribosomes on the biotinylated RNA. Following tethering of the complex, molecules were imaged at equilibrium using TIRFM. Molecules were expected to begin in a Cy3 (green, FRET donor) to Cy5 (red, acceptor) FRET state, followed by photobleaching of both dyes. The region of the trace that corresponds to FRET is highlighted by the gray box. (*C*) Plot of the distribution of observed FRET efficiencies for the intersubunit FRET signal on 80S ribosomes. Frequencies of observed FRET efficiencies were binned into 35 bins (open circles) across the indicated range. The line represents a fit to a single-Gaussian function, which yielded a mean FRET efficiency of 0.5 ± 0.01 (95% CI); *n* = 104. (*D*) Schematic of the single-molecule fluorescence assay. The 40S ribosomal subunits were labeled with Cy3 dye via uS19-ybbR, and 60S subunits were labeled with Cy5 via uL18-ybbR. Preformed 80S–CrPV IRES complexes were tethered to the ZMW imaging surface. At the start of data acquisition, Cy5.5-NSP1 (N-terminal ybbR tag) was added at 25 nM (final concentration) at 30 °C. (*E*) Example single-molecule fluorescence traces that depict addition of Cy5.5-NSP1 to tethered CrPV+1 RNAs bound by 80S ribosomes. The 40S subunit was labeled with Cy3 (green), 60S subunit with Cy5 (red), and NSP1 with Cy5.5 (magenta). The two traces are from the same experiment where 80S–CrPV+1 complexes were tethered. *Top* trace depicts a complex with an NSP1 binding event (∼30% of traces), and *Bottom* trace lacks an NSP1 event (∼70% of traces). Raw fluorescence intensities were corrected in this image to set baseline intensities to zero for presentation. Due to bleed through across the three fluorescent channels, the Cy3, Cy5, and Cy5.5 signals were made transparent before and after relevant events for presentation here. The association time (Δ*t*) was defined as the time elapsed from the addition of Cy5.5-NSP1 until the burst of Cy5.5 fluorescence (magenta), which signified NSP1 association. The lifetime was defined as the duration of the Cy5.5 fluorescence signal. (*F*) Plot of the cumulative probability of observed NSP1 association times with the indicated ribosomal–CrPV IRES complexes at 30 °C. Cy5.5-NSP1 was added at 25 nM (final concentration). Lines represent fits to double-exponential functions. See *SI Appendix*, Table S5 for samples sizes and the parameters for relevant fits.

Intriguingly, NSP1 has been visualized bound to 80S ribosomal complexes isolated from cellular extracts (19, 20). To examine whether NSP1 could associate with 80S ribosomes, we used CRISPR-Cas9 and homology-directed repair to establish a Förster resonance energy transfer (FRET) signal between the 40S and 60S subunits of the ribosome, analogous to our signal to track 80S ribosome formation in yeast (43). The ybbR tag was appended to all endogenous copies (HEK293T cells) of ribosomal proteins uS19 (40S subunit) or uL18 (60S subunit) (*SI Appendix*, Fig. S6 *A*–*D*), which are within predicted FRET distance (∼50 Å) in structural models of 80S ribosomes (Fig. 5*A*). The tagged ribosomes were functional in cells (*SI Appendix*, Fig. S6*E*), and purified 40S-ybbR and 60S-ybbR subunits were labeled efficiently (50 to 80%) with Cy3 (FRET donor) and Cy5 (FRET acceptor) fluorescent dyes, respectively (*SI Appendix*, Fig. S6*F*). After incubation with the IRES from the intergenic region of cricket paralysis virus (CrPV IRES), which assembles ribosomal subunits into 80S ribosomes independent of eIFs (44), we observed a FRET efficiency distribution (mean≈0.5±0.01, 95% CI) between the labeled 40S and 60S subunits, consistent with structural predictions (Fig. 5 *B* and *C*).

By leveraging the FRET signal and the CrPV IRES, we examined whether NSP1 associated with 80S ribosomes assembled on an mRNA (Fig. 5*D*). We generated RNAs as above with 1, 6, and 48 nt downstream of the CCU codon present in the ribosomal A site (*SI Appendix*, Fig. S7 *A* and *B*). With these models, the RNA is shifted 3 nt farther into the entry channel relative to the HCV IRES (45). Therefore, CrPV+6 and CrPV+48 will have mRNA that at least partially occludes the NSP1 binding site, whereas CrPV+1 will not. When added at 25 nM to 40S–CrPV+1 complexes, we observed slower (median Δ*t* ≈ 119 s to 189 s) and less efficient (45 ± 4%) Cy5.5-NSP1 association relative to that of 40S–HCV+0 complexes (median Δ*t* ≈ 46 s to 82 and 72 ± 4%) (*SI Appendix*, Fig. S7 *C* and *D* and Table S5). This finding very likely reflects heterogeneity of the 40S subunit head conformation when bound to the CrPV IRES (46), unlike the near-homogenous open conformation induced by the wild-type HCV IRES. Further inclusion of 60S subunits to yield 80S–CrPV+1 complexes inhibited NSP1 association (median Δ*t* ≈ 235 s to 285 s), similar to the inhibition observed on both 80S–CrPV+6 and 80S–CrPV+48 complexes (median Δ*t* ≈ 243 s to 292 s and 288 s to 354 s) (Fig. 5 *E* and *F* and *SI Appendix*, Fig. S7*D*). Thus, even when mRNA was absent from it, the conformation of the mRNA entry channel on 80S–CrPV IRES complexes was incompatible with rapid NSP1 association. Whether NSP1 accesses other states of the 80S ribosome and how visualized NSP1–80S complexes (19, 20) form require further investigation.

### NSP1 Remained Bound to 40S Subunits upon Association with Model mRNAs.

**Fig. 6.**
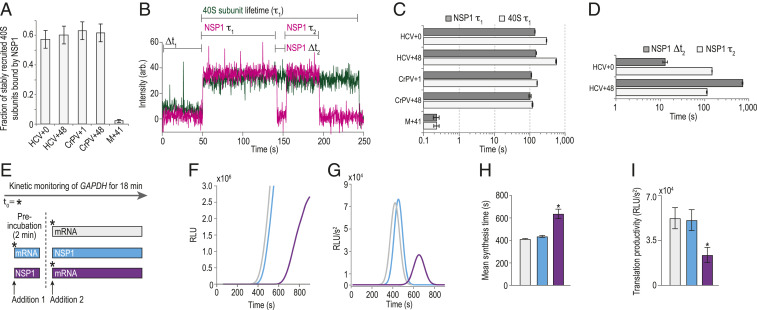
NSP1 remained bound to 40S subunits upon association with model mRNAs. (*A*) Plot of the fraction of stable (≥5 s) 40S subunit binding events that coassociated with NSP1, as defined in *B*. Error bars represent 99% CI. (*B*) Example single-molecule fluorescence trace that depicts association of NSP1–40S subunit complexes with a tethered HCV+0 IRES molecule. The 40S subunit and NSP1 were labeled with Cy3 (green) and Cy5.5 (magenta) dyes, respectively. Raw fluorescence intensities were corrected in this image to set baseline intensities to zero for presentation. The initial NSP1–40S subunit association time (Δ*t*_1_) was defined as the time elapsed from the addition of the complex until the burst of Cy3 and Cy5.5 fluorescence, which signified association of the NSP1-40S subunit complex with the tethered IRES. In experiments that lacked NSP1, Δ*t*_1_ was defined using the first burst of Cy3 signal alone. The 40S subunit lifetime $\left(\tau_{1}\right)$ was defined as the duration of the Cy3 fluorescence signal. The initial NSP1 lifetime $\left(\mathrm{NSP}1\;\tau_{1}\right)$ was defined as the duration of the Cy5.5 signal that coappeared with the Cy3 signal. For NSP1 reassociation analyses, we focused on ZMWs where a single 40S subunit associated within the first 200 s (∼75% of all events; see *SI Appendix*, Fig. S8*F*). We then quantified the time elapsed from the loss of the first Cy5.5 signal to the next burst of Cy5.5 fluorescence at least ∼20 s in length (∼70% of initial NSP1 binding events), which was defined as the NSP1 reassociation time (NSP1 Δ*t*_2_). The duration of this second Cy5.5 event was defined as the reassociated NSP1 lifetime $\left(\mathrm{NSP}1\;\tau_{2}\right)$. (*C* and *D*) Plots of the indicated lifetimes. Here, all experiments were conducted in the presence of NSP1. See *SI Appendix*, Fig. S8 *D* and *E* for 40S subunit lifetimes in the absence of NSP1. Lifetimes were defined as the reciprocal of the predominate dissociation rate derived from fits to double-exponential functions, with error bars representing the 95% CI of the rate. See *SI Appendix*, Table S6 for samples sizes and the parameters for relevant fits. (*E*) Time-of-addition cell-free IVT experimental design. GAPDH reporter mRNA and WT NSP1 (400 nM) were added to HeLa IVT reactions in the order depicted above. The nLuc signal was continuously monitored in situ. Using the same color scheme as *E*, *F*–*I* depict results from six independent replicates for each experimental condition, except for the “preincubation with NSP1 reaction” (red bar in *E*), which has *n* = 3. (*F*) Time course of nLuc synthesis from a representative time-of-addition experiment. (*G*) Representative Gaussian fits of the second derivative of nLuc synthesis time course data shown in *F*. (*H*) Plot of mean synthesis time. Error bars represent SEM, *P < 0.0001. (*I*) Plot of translational productivity. Error bars represent SEM, *P = 0.045, one-way ANOVA.

While accommodated mRNA inhibited NSP1 association, it remained unclear whether mRNA could destabilize the NSP1–40S subunit complex upon its recruitment. Using Cy5.5-NSP1 and 40S-Cy3 subunits, we preformed NSP1–40S complexes and added the complex at 15 nM to ZMWs with surface-immobilized model mRNAs (*SI Appendix*, Fig. S8 *A*–*C*). On HCV+0 and HCV+48, NSP1 coassociated with 57 ± 6% and 60 ± 6% of recruited 40S subunits (Fig. 6 *A* and *B* and *SI Appendix*, Table S6), which indicated near-saturation of 40S subunits with NSP1. Association of the NSP1–40S subunit complex with these tethered RNAs had kinetics similar to 40S subunits alone (*SI Appendix*, Fig. S8 *D*–*F*), as expected given the high-affinity IRES–40S subunit interaction independent of the mRNA cleft. After association, NSP1 remained bound to both complexes for ∼120 s $\left(k_{off}\approx 0.0072\pm 0.002\;s^{-1}\text{ and }0.0067\pm 0.0001\;s^{-1}\right)$ (Fig. 6*C* and *SI Appendix*, Fig. S8*F*). Similarly, NSP1 was long lived on the ribosomal subunit after recruitment to CrPV+1 and CrPV+48 model RNAs $\left(k_{off}\approx 0.0091\pm 0.0002\;s^{-1}\text{ and }0.0099\pm 0.0008\;s^{-1}\right)$ (Fig. 6*C* and *SI Appendix*, Fig. S8 *D*, *E*, and *G*–*I*). In stark contrast, NSP1–40S subunit preinitiation complexes (eIF1, eIF1A, eIF5, TC-GMPPNP) corecruited to M+41 model mRNAs had very short lifetimes $\left(k_{off}\approx 4\pm 1\;s^{-1}\right)$, rapidly codeparting from the mRNA after its expected slow association (Fig. 6*C* and *SI Appendix*, Fig. S8 *D*, *E*, and *J*–*L*). Unlike the IRESs, the presence of NSP1 on the 43S PIC likely prevented a stable interaction with M+41 by blocking its accommodation into the entry channel in the absence of the stabilizing m^7^G cap–eIF4F–eIF3–40S network of interactions. Indeed, 43S PICs that stably associated with M+41 mRNAs $\left(k_{off}\approx 0.0038\pm 0.0002\;s^{-1}\right)$ were depleted about 30-fold for NSP1 (2 ± 1%) (Fig. 6*A* and *SI Appendix*, Fig. S8 *E*, *J*, and *L*).

To further delineate competition between NSP1 and mRNA for the 40S entry channel, we asked whether NSP1 could reassociate stably with single HCV IRES–40S subunit complexes and how reassociation was impacted by long segments of RNA downstream of the start codon. Following loss of the initial NSP1 signal (due to dye photobleaching or NSP1 departure), 80 ± 6% of 40S–HCV+0 complexes had at least one additional stable (≥20 s) NSP1 binding event (*SI Appendix*, Fig. S8 *B* and *M*). In contrast, only 32 ± 7% of 40S–HCV+48 complexes had a second, stable NSP1 event (*SI Appendix*, Fig. S8 *C* and *M*). When multiple NSP1 association events were observed on a single 40S–IRES complex, the NSP1 reassociation rate was at least 55-fold slower on HCV+48 $\left(k_{obs}\leq ∼0.0014\;s^{-1}\right)$ relative to HCV+0 $\left(k_{obs}\approx 0.078\pm 0.01\;s^{-1}\right)$ (Fig. 6*D* and *SI Appendix*, Fig. S8*N* and Table S6), which had association kinetics similar to the protein with the apo complex (Figs. 3 and 4). The lifetimes of initial and reassociated NSP1 binding events were similar (Fig. 6*D* and *SI Appendix*, Fig. S8*O*). Together, these findings indicated that, once NSP1 dissociated from the 40S–HCV+48 complex, mRNA was accommodated more rapidly into the mRNA entry channel, thereby inhibiting reassociation of NSP1.

Our single-molecule findings indicated that the presence of NSP1 and mRNA are mutually exclusive in the entry channel of the 40S subunit. We therefore hypothesized that ribosomes preassembled on an mRNA could evade NSP1-mediated translation inhibition. Using real-time IVT assays, we either preincubated extracts with NSP1 (400 nM) or mRNA (80 nM) prior to addition of the other component (Fig. 6*E*). Preincubation of extracts with NSP1 prior to mRNA delayed the appearance of nLuc signal, but not vice versa (Fig. 6*F*). To quantitate this difference, we fit the second derivative of the time course data to a Gaussian distribution (Fig. 6*G*) (47). As suggested by the raw data, the mean synthesis time (Gaussian mean) when extracts were preincubated with NSP1 (637 ± 41 s) increased by 54% (*P* < 0.0001, one-way ANOVA) compared to the reaction without NSP1 (413 ± 5 s) (Fig. 6*H*). This lag was similar in length to our best estimate for the lifetime of NSP1 on the 40S subunit (≥250 s). Preincubation with NSP1 also reduced translational productivity (Gaussian amplitude) approximately twofold (*P* = 0.04, one-way ANOVA) (Fig. 6*I*), similar to when NSP1 and mRNA were added simultaneously in endpoint assays (Fig. 1). In contrast, preincubation with mRNA yielded mean synthesis times and translation productivity similar to reactions that lacked NSP1 (Fig. 6 *H* and *I*). Thus, the impact of NSP1 on translation was dependent on its time of addition to the IVT reaction, which suggests that mRNAs preloaded on ribosomes can evade NSP1-mediated inhibition.

## Discussion

**Fig. 7.**
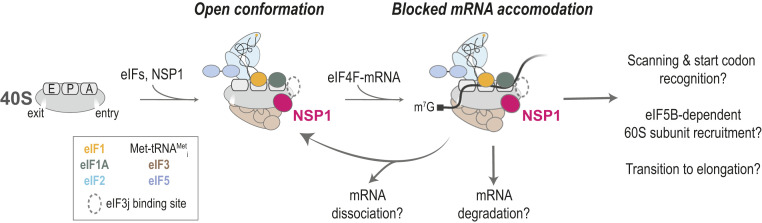
Proposed model. In the absence of eIF3j, NSP1 preferentially associates with the open conformation of the 40S subunit to block full accommodation of the mRNA in the entry channel, which inhibits translation initiation. How incomplete mRNA accommodation impacts mRNA recruitment, scanning, start codon selection, 60S subunit recruitment, and the transition to elongation remain open questions. Whether and how NSP1–40S subunit complexes lead to mRNA degradation is also unknown.

Shutdown of host protein synthesis is a common feature of viral infection. Most characterized mechanisms involve the covalent inactivation of key eIFs or their regulators [e.g., eIF2 and eIF4F (48)]. Here, we provide insight into a distinct form of translation inhibition employed by SARS-CoV-2 and other beta-CoVs. The first protein encoded in the viral genomic RNA, NSP1, directly targets the small subunit of the human ribosome to inhibit protein synthesis. Based on our findings and recent structural studies (19, 49), we suggest that NSP1 preferentially associates with the open conformation of the 40S subunit to prevent proper accommodation of mRNA during translation initiation (Fig. 7).

NSP1 is a potent inhibitor of human translation. When purified NSP1 was added to human HeLa cell extract, we observed a strong reduction in translation of our model for human GAPDH mRNA. Inhibition was specific; mutations in two NSP1 amino acids (KH164-165) necessary for 40S subunit binding abrogated the activity. The apparent IC_50_ for NSP1-mediated inhibition suggests near-stoichiometric association of NSP1 with 40S subunits in the cell extract, which agrees well with our best estimate for the *K*_*D*_ of the interaction (≤1 nM). Similarly, NSP1 inhibited translation of SARS-CoV-2 model mRNAs at levels comparable to that for the model human mRNA. Together, these findings suggest that NSP1 is a general inhibitor of human protein synthesis. Upon infection, the high affinity of NSP1 for the 40S subunit likely requires buildup of NSP1 protein concentration before translation is inhibited broadly, which may enable viral protein synthesis to proceed unimpeded during early stages. Once NSP1 has accumulated, the increased translation efficiency of the viral mRNAs relative to human mRNAs we and others (49) have observed may enable the virus to synthesize sufficient amounts of viral proteins, even when translation is largely shut down. However, our assays were performed in cellular extracts with model mRNAs in the absence of SARS-CoV-2 infection. It therefore is plausible that a missing cellular or viral protein, a segment of the viral genome, or another mechanism (e.g., sequestration) further allows the virus to evade translation inhibition. Future studies in the context of infected cells are needed to deconvolute these possibilities.

NSP1 preferentially associates with the open conformation of the 40S subunit. The most rapid NSP1 association with the 40S subunit we observed was in the presence of the HCV IRES. This structured RNA directly manipulates the ribosomal subunit to bypass eIFs and initiate translation (50). One of its flexible segments, domain II, makes extensive contacts with the 40S subunit head, which swivels, opening the mRNA entry channel (38–40). In our assays, the estimated rate of NSP1 association with 40S subunits bound to the HCV+0 model mRNA was 3 µM^−1^⋅s^−1^ to 4 µM^−1^⋅s^−1^, nearly an order of magnitude faster than with ribosomes alone. Upon removal of IRES domain II, ribosomal association of NSP1 was inhibited at a level similar to that of the KH164-165AA mutant and MERS-CoV NSP1 proteins, both of which lack ribosome binding activity. The conformational shift of the complex into the closed state in the absence of IRES domain II thus largely blocked NSP1 binding. Consistent with this interpretation, NSP1 associated more slowly with the 40S–CrPV+1 complex, which contains a heterogenous mix of open and closed entry channel conformations (46). Of all eIFs we examined, NSP1 association was enhanced the most (approximately twofold) by eIF1, which binds with high affinity to the ribosomal P site (51–53) and has a critical role during start codon recognition (54–58). Given that eIF1 and NSP1 binding sites are nonoverlapping (*SI Appendix*, Fig. S3*L*), our findings demonstrate that eIF1 allosterically enhances NSP1 association, likely by altering the conformation of the mRNA entry channel.

NSP1 dynamically competes with mRNA to bind the ribosome. When 40S subunits were preincubated with mRNA that had at least 12 nt downstream of the start codon, we observed marked inhibition of NSP1 association with the ribosomal subunit. On such mRNAs, the NSP1 binding site within the entry channel is occluded by the accommodated mRNA. Yet, NSP1 remained bound to the 40S ribosomal subunit upon recruitment of an mRNA—regardless of its length. This finding suggests that mRNA itself is insufficient to dislodge or destabilize the NSP1–40S subunit interaction. However, once NSP1 dissociated, mRNA was accommodated into the entry channel, which prevented reassociation of the protein. Similarly, NSP1 association with the 40S subunit was inhibited by preincubation with eIF3j, which binds the 40S subunit with high affinity (31, 36). Much like NSP1, the C terminus of eIF3j binds anticooperatively with mRNA in the entry channel of the 40S subunit in the absence of other eIFs (31, 32, 59). While it was unresolved in a recent high-resolution structure of a 48S preinitiation complex (33), this region of eIF3j may sterically block NSP1 and/or mRNA association (31). Alternatively, eIF3j may limit movement of the 40S subunit head through its contacts with the mRNA entry latch (rRNA helix 34) (33) to promote an entry channel conformation inaccessible to both, perhaps the closed head conformation (60). Regardless, our findings collectively indicate that NSP1 and mRNA are unable to cooccupy the mRNA entry channel of the 40S subunit.

The presence of NSP1 in ribosomal preinitiation complexes very likely prevents accommodation of mRNA into the entry channel of the 40S subunit to inhibit translation initiation. Consistent with this model, we found that NSP1–40S preinitiation complexes were unable to bind stably to a simple model mRNA, unlike complexes that lacked the protein. Moreover, when we preincubated NSP1 in extracts prior to mRNA addition, there was a marked delay prior to detectable protein synthesis (∼220 s). This lag was similar in length to the observed lifetime of the NSP1–40S subunit interaction (at least ∼250 s) and longer than the time frame of translation initiation on many mRNAs (<60 s) (61, 62), the presumptive rate-limiting phase of protein synthesis (26, 61). We reasoned that, if the NSP1-induced delay was due to incomplete or improper mRNA loading during translation initiation—as suggested by our single-molecule assays—preloading the mRNA into the ribosome may evade inhibition. Indeed, preincubation of extracts with mRNA eliminated NSP1-mediated translation inhibition in our in vitro translation assays. While NSP1 may impact other phases of protein synthesis (63), our collective findings strongly suggest that the protein is a potent inhibitor of translation initiation. Agreeably, ectopic expression of NSP1 in cells reduced the abundance of actively translating polysomes and increased the abundance of 80S monosomes (16, 17, 19), hallmarks of disrupted initiation.

Our work provides a biophysical foundation for NSP1-mediated shutdown of host translation and SARS-CoV-2 pathogenicity. Nevertheless, gaps remain in the molecular model for how the protein disrupts each step in the highly coordinated process of translation initiation. While they provided powerful advantages to probe NSP1 function, the model mRNAs we leveraged employ translation initiation strategies divergent from many human mRNAs and function in the absence of eIFs (CrPV) or with a subset (HCV, M+41) that can be alternatively positioned [eIF3 on HCV (64)]. Instead of direct recruitment, human mRNAs typically rely on interactions between the m^7^G cap, eIF4F, and the ribosomal preinitiation complex to initiate translation, involving RNA helicases such as eIF4A, DDX3X, and DHX29. They also may utilize multiple modes of mRNA recruitment [e.g., “slotting” (33) versus “threading” (65, 66)] and mechanisms (e.g., scanning) not reflected well by our model mRNAs and limited set of eIFs. Future studies that build from our foundation and the single-molecule tools debuted here will delineate how NSP1 impacts eIF4F-dependent and alternative paths of translation initiation, and illuminate how mRNA accommodation is coupled to scanning, start codon recognition, and other dynamic steps of the process.

## Materials and Methods (Condensed)

Please see *SI Appendix* for a detailed version of all materials and methods.

### Molecular Cloning.

See Dataset S1 for all relevant sequences. Codon-optimized NSP1 proteins and relevant mutants were cloned into a vector purchased from the University of California, Berkeley QB3 MacroLab (vector 1B), which encoded an N-terminal 6xHis tag. When noted, a ybbR tag was included on either the N terminus (ybbR-NSP1) or the C terminus (NSP1-ybbR). For the IRESs, a synthetic DNA was purchased from Integrated DNA Technologies (IDT) that encoded the HCV IRES, and a plasmid that encodes the IRES of the intergenic region of CrPV with the first codon (Ala) replaced with a Phe codon (TTC) was described previously (67). For nLuc reporters, the nLuc coding sequence (Promega) flanked by the 5′ and 3′ UTRs from human GAPDH (National Center for Biotechnology Information [NCBI] GenBank accession: AF261085), and a poly(A) tail. Viral DNA (NCBI GenBank accession MN997409.1) constructs were designed such that the nLuc coding sequence was flanked by either the full-length 5′ UTR or the subgenomic 5′ LDR and one of two 3′ UTR sequences, and a poly(A) tail.

### NSP1 Expression, Purification, and Labeling.

All NSP1 proteins were expressed in OneShot BL21(DE3) cells (Invitrogen). Cells were lysed by sonication in lysis buffer (20 mM Tris⋅HCl pH 8.0, 300 mM NaCl, 10% [vol/vol] glycerol, 40 mM imidazole, and 5 mM β-mercaptoethanol). Clarified lysate was loaded onto a Ni-nitrilotriacetic acid (Ni-NTA) gravity flow column equilibrated in lysis buffer, washed with 20 column volumes (CV) of lysis buffer, 20 CV of wash buffer (20 mM Tris⋅HCl pH 8.0, 1 M NaCl, 10% [vol/vol] glycerol, 40 mM imidazole, and 5 mM β-mercaptoethanol), and 10 CV of lysis buffer. Recombinant proteins eluted in elution buffer (20 mM Tris⋅HCl pH 8.0, 300 mM NaCl, 10% [vol/vol] glycerol, 300 mM imidazole, and 5 mM β-mercaptoethanol). Relevant fractions were dialyzed overnight at 4 °C into ybbR-labeling buffer (50 mM Hepes-KOH pH 7.5, 250 mM NaCl, 10 mM MgCl_2_, 10% [vol/vol] glycerol, and 1 mM dithiothreitol [DTT]) or Tobacco Etch Virus (TEV) Protease Cleavage Buffer (20 mM Tris⋅HCl pH 8.0, 250 mM NaCl, 10% [vol/vol] glycerol, 10 mM imidazole, and 5 mM β-mercaptoethanol), as appropriate. Fluorescent labeling via the ybbR tag was performed essentially as described (29, 34). Following cleavage, TEV protease, Sfp synthase, and the cleaved 6His tag were removed via a subtractive Ni-NTA gravity column equilibrated in TEV buffer. NSP1 proteins were subjected to a final purification step using size exclusion chromatography (SEC) on a Superdex 75 column (23 mL) equilibrated in SEC buffer (20 mM Hepes-KOH pH 7.5, 250 mM KOAc, 10% [vol/vol)] glycerol, and 1 mM DTT). Fractions containing NSP1 were concentrated using a 10-kDa MWCO Amicon Ultra centrifugal filter, aliquoted, flash frozen on liquid N_2_, and stored at −80 °C. For ybbR-NSP1, labeling efficiencies were 50 to 70%. For NSP1-ybbR, the labeling efficiency was much lower (<20%), and the protein had reduced translation inhibition activity, which is why it was excluded from single-molecule analyses.

### nLuc In Vitro Translation Assays.

HeLa cell-free translation (ThermoFisher, #88882) reactions set up according to manufacturer’s protocol were programmed with a final mRNA concentration of 200 nM (endpoint) or 80 nM (real time). Endpoint assays were incubated at 37 °C for 45 min. Real-time assays were prepared by addition nGlow substrate to the cell-free translation mix with a 1:10 vol/vol ratio. Before the addition of mRNA and/or NSP1, the IVT reactions were transferred to nonadjacent wells in a 384-well plate and equilibrated to 30 °C in the plate reader. All other reagents were maintained at 30 °C and then added to the IVT reactions according to the order-of-addition assay schematic outlined in Fig. 6*E*. The preincubation (30 °C, 2 min) was performed in the plate reader. Kinetic monitoring of the samples (36 min, 15-s intervals) was initiated during the equilibration step. Data were analyzed in MatLab using the approach developed by Vassilenko et al. (47).

### Native Gel Assays.

Native composite agarose/acrylamide gels were prepared as described (68). For complex formation, the indicated components were incubated in ribosome assay buffer (30 mM Hepes-KOH pH 7.4, 100 mM KOAc, 2 mM Mg(OAc)_2_) at 37 °C for 15 min. Unless noted, NSP1 with a C-terminal ybbR tag conjugated to a Cy5 dye was used in all gel shift experiments, as C-terminally tagged NSP1 from SARS-CoV was functional in cellular assays (12) and Cy5 provides cleaner signal upon image acquisition with a Typhoon imager. For competition experiments, the competitor protein was preincubated with ribosomal subunits at 37 °C for 15 min, prior to addition of the labeled protein.

### Purification of Human eIFs and tRNA_i_.

The eIF1 (31), eIF1A (31), eIF2 (31, 69), eIF3 (31, 69), and eIF5 (70) protein purifications were performed as described. The eIF3j was expressed and purified as done for NSP1, with the changes noted in *SI Appendix*. Human tRNA_i_ was in vitro transcribed from a DNA template with a 5′-end T7 promoter and hammer head ribozyme (31), during which the ribozyme self-cleaved (>80% efficiency). Mature tRNA_i_ was separated from precursor RNA and cleaved ribozyme RNA via 10% acrylamide gel electrophoresis in the presence of 8 M urea. After band extraction, tRNA_i_ was resuspended in 10 mM NaCl, 10 mM Bis-Tris, pH 7.0 and stored at −80 °C. The tRNA_i_ was charged with l-methionine using yeast MetRS (71). The resulting tRNA was purified by phenol/chloroform/isoamyl alcohol (25:24:1, pH 5.2) extraction and ethanol precipitation. The pellet was resuspended with tRNA storage buffer (10 mM NaOAc pH 5.2, 50 mM Mg(OAc)_2_) and further purified by passing through BioRad P-6 columns that were equilibrated with tRNA storage buffer. The charging efficiency was ∼70%, based on acid urea PAGE analyses (72) of the final tRNA product.

### Real-Time Single-Molecule Assays Using ZMWs.

All real-time imaging was conducted using a modified Pacific Biosciences RSII DNA sequencer (35). Unless noted, Cy3 dyes were excited using the 532 nm excitation laser at 0.6 µW/µm^2^. Cy5 and Cy5.5 dyes were excited with the 642 nm laser at 0.1 µW/µm^2^. In nearly all experiments, data were collected at 10 frames per second. The exception was when Cy3 was excited with the 532 nm laser at 0.16 or 0.1 µW/µm^2^ to enhance dye stability and data were collected at 3 fps to increase signal to noise ratios. ZMW chips were purchased from Pacific Biosciences. In all real-time experiments, fluorescently-labeled NSP1 with an N-terminal ybbR tag was used, since it had translation inhibition and 40S-binding activities similar to the wild-type protein. Please see *SI Appendix*, *Methods and Methods (Expanded)* for all experimental conditions.

### Data Analysis.

Experimental movies that captured fluorescence intensities over time were processed in MATLAB as described previously (34, 35). Unless noted, only the first NSP1 binding event longer than ∼5 s that occurred within the first 500 s of imaging was analyzed. Unless intractable, ∼1,000 molecules were analyzed to determine binding efficiencies, and 200 single molecules were analyzed for kinetic analyses, indicated by single-step photobleach events. Association times were defined as the time elapsed from the addition of the labeled component until a burst of fluorescence for that component. The time of addition is controlled by the instrument and varies, but typically occurs within the first 10 s of data acquisition and is accounted for in the analyses. Lifetimes were defined as the duration of the corresponding fluorescence signal.

Kinetic parameters were extracted by fitting observed data to single- or double-exponential functions as described (35). On some complexes, the presence of a large, slow association phase made it difficult to derive reliable rates, as amplitudes for the association rates are assigned semiarbitrarily during the fits. When this occurred, comparisons of median association times were used instead, which better reflected the raw data. In *Results*, this is indicated by “median association times,” which only pertains to the indicated final concentration of NSP1. All derived association rates, median association times, lifetimes, and the number of molecules examined are reported in *SI Appendix*, Tables S2–S6. As indicated in the tables, fits to linear functions were used to estimate very slow association rates observed when NSP1 association was inhibited. To calculate errors for NSP1 binding efficiency (e.g., Fig. 3*E*), bootstrap analyses (*n* = 10,000) were performed to calculate 99% CI for the observed proportions using R. To calculate errors for median association times and lifetimes, bootstrap analyses (*n* = 10,000) were performed to calculate 95% CI of the observed median using MATLAB. Reported errors for derived rates represent 95% CI.

### Ribosome Purifications and Labeling.

The 40S and 60S ribosomal subunits were purified from the indicated cell lines and labeled with biotin or dyes as described (34).

### CRISPR-Cas9 and Homology-Directed Repair.

To generate the 40S-uS19-ybbR and 60S-uL18-ybbR cell lines, guide sequences were cloned into pX458 using the published approach (73). To insert the tandem ybbR and flag tags onto the endogenous copies of the genes, single-stranded DNA ultramer repair templates were purchased from Integrated DNA Technologies that contained about 40 nt to 60 nt of flanking sequence on either side of the desired insertion. See Dataset S1 for all guide oligo, repair template, and PCR screening oligo sequences. Approximately 24 h post seeding in a well of a six-well plate, low-confluency (∼30%) wild-type HEK293T cells were transiently transfected (Liopfectamine 3000, ThermoFisher) with 1 µg of the relevant pX458 plasmid and 2 µg of single-stranded DNA repair template. Cells recovered for 48 h. Single, eGFP-positive cells were sorted at the Stanford Shared Fluorescence-activated cell sorting (FACS) Facility into a well of a 96-well plate that contained 50% conditioned Dulbecco’s modified Eagle’s medium (high glucose). Individual colonies recovered until they were visible by eye. Colonies then were transferred to a well of a 24-well plate and screened via PCR, Sanger sequencing, and Western blot analyses.

### Structural Models.

All structural models were rendered using ChimeraX (74). The following Protein Data Bank (PDB) models were used: PDB ID codes 4UG0 (75), 5A2Q (38), 6ZLW (19), 6YAL (76), and 6ZMW (33).

## Supplementary Material

Supplementary File

Supplementary File

## Data Availability

Some study data are available.
