# Supplementary material for: Dynamic competition between SARS-CoV-2 NSP1 and mRNA on the human ribosome inhibits translation initiation
Source: Proc Natl Acad Sci U S A. 2021 Jan 21;118(6):e2017715118. doi: 10.1073/pnas.2017715118 (PMC8017934; doi:10.1073/pnas.2017715118)
Supplement: Supplementary File [file pnas.2017715118.sapp.pdf]

SI APPENDIX

Supplementary Figures 1-8

Supplementary Tables 1-6

Expanded Material and Methods

Supplementary References

SUPPLEMENTARY FIGURES

Supplementary Figure 1

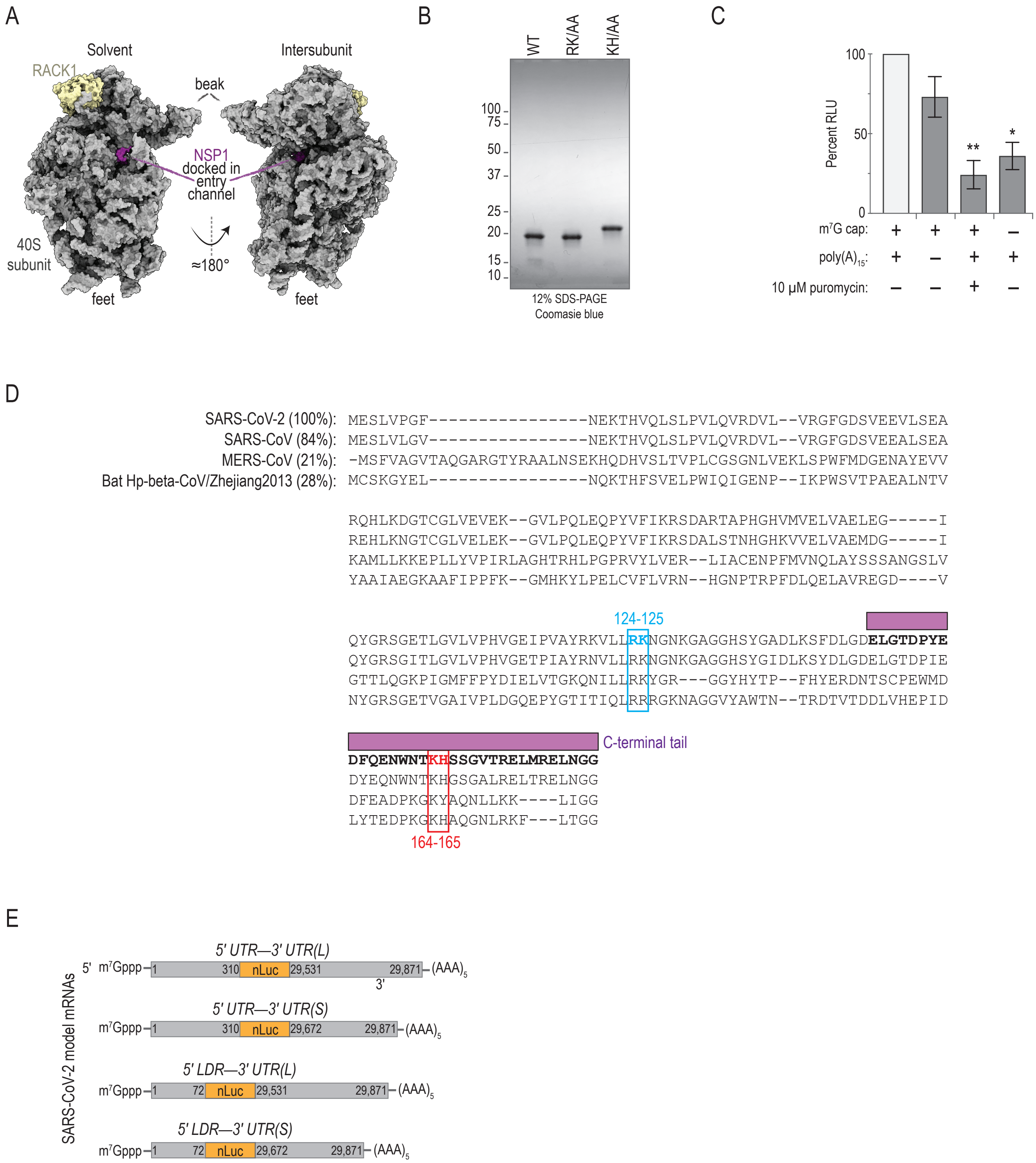

Figure S1. NSP1 from SARS-CoV-2 inhibited translation initiation. Related to **Fig. 1.**

A). Model of NSP1 bound to the mRNA entry channel of the human 40S subunit (PDB:6ZLW)**.** The 40S subunit is depicted in gray, RACK1 in light yellow, and NSP1 in purple.

B). Image of a gel that depicts recombinantly-expressed and purified SARS-CoV-2 NSP1 proteins examined using SDS-PAGE on a 12% acrylamide gel. The NSP1 mutant protein with KH164-165 substituted to alanine (KH/AA) runs at a different apparent molecular weight relative to the wild-type (WT) or RK124-125 to alanine (RK/AA) mutant.

C). Plot of the mean nLuc signal from cell-free translation of GAPDH reporter mRNAs in the indicated conditions (n = 3). Mean translational activity (percent RLU) of GAPDH mRNA was reduced significantly (one-way ANOVA, p-value $\leq$ 0.002) in the absence of an m^7G cap and more modestly by the lack of a poly(A) tail, key features of protein synthesis in cells. Inclusion of puromycin significantly inhibited protein synthesis (one-way ANOVA, p-value $\leq$ 0.0008), as expected. Error bars represent SEM.

D). Alignment of NSP1 protein sequences from SARS-CoV-2, SARS-CoV, MERS-CoV, and bat-Hp CoV from the Hibecovirus subgenus (Bat Hp-beta-CoV/Zhejiang2013). The percent sequence identities are indicated (relative to SARS-CoV-2). The location of substitutions predicted to disrupt NSP1-mediated destabilization of mRNAs (RK124-125, blue box) and 40S-binding activities (KH164-164, red box) are highlighted. Based on recent structural studies, the C-terminal amino acids that dock within the mRNA entry channel of the 40S subunit are bolded and highlighted by the purple box. NCBI GenBank accessions are, respectively: MN997409.1, AY508724, JX869059, and KF636752.

E). Schematic of SARS-CoV-2 nLuc reporter mRNAs used in HeLa cell-free *in vitro* translation (IVT) assays. The nLuc coding sequence was flanked by either the full-length viral 5'UTR or the subgenomic 5' leader sequence (LDR) and either the 3'UTR(L), which contains part of N ORF or 3'UTR(S), which begins after ORF10. Numbers refer to nucleotide position in the viral genome (NCBI GenBank accession: MN997409.1).

Supplementary Figure 2

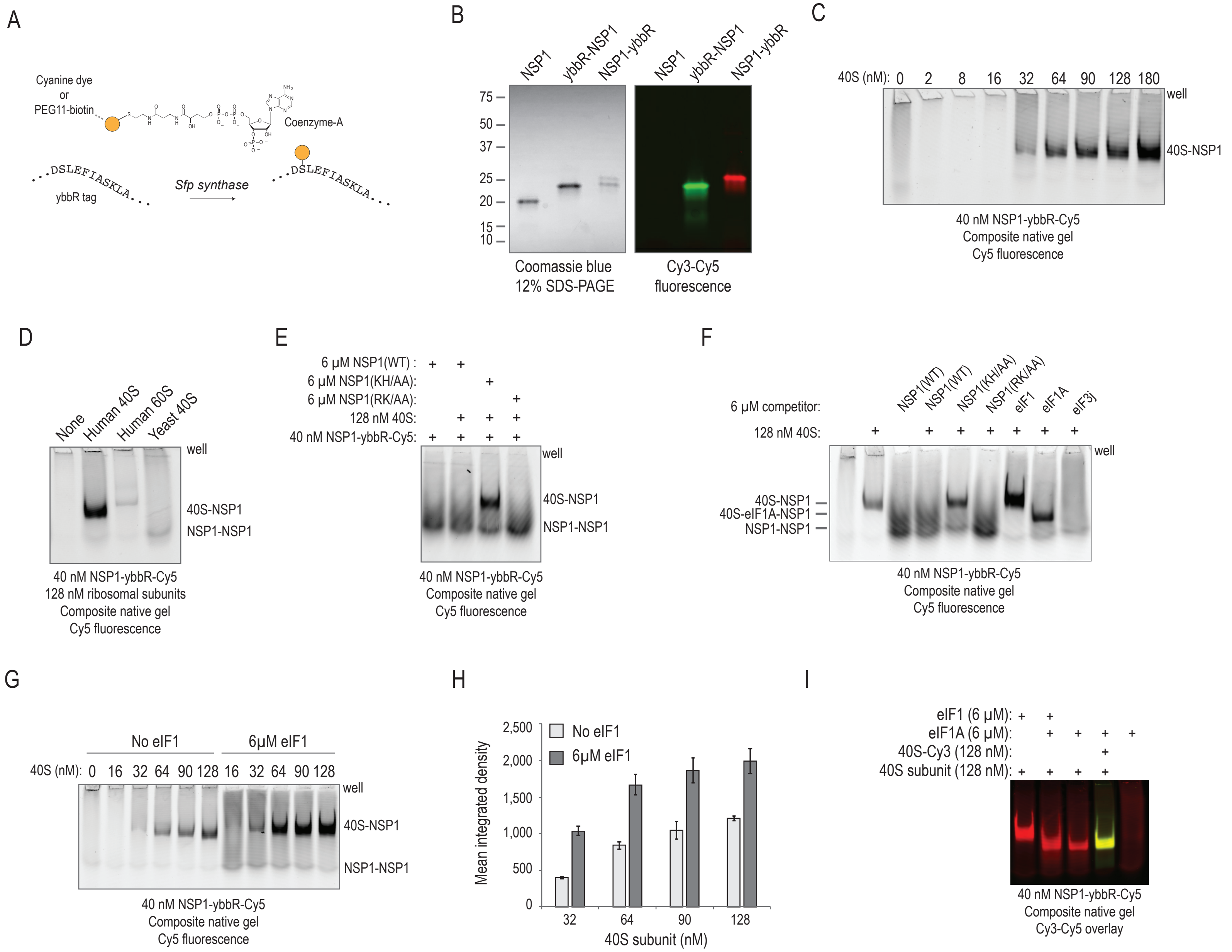

Figure S2. NSP1 specifically bound the human 40S ribosomal subunit, which was enhanced by eIF1 and blocked by eIF3j. Related to **Fig. 2.**

A). Schematic of site-specific, enzymatic attachment of fluorescent cyanine dyes or biotin to the ybbR peptide tag by Sfp synthase.

B). Image of purified and fluorescently-labeled NSP1 analyzed via SDS-PAGE. N-terminally tagged 'ybbR-NSP1' was labeled with Cy3 fluorescent dye, and C-terminally tagged 'NSP1-ybbR' was labeled with Cy5 dye. The image depicts total protein stained with Coomassie blue (left) after scanning the gel for Cy3 and Cy5 fluorescence (right), shown as an overlay of the fluorescent signals. The doublet in the NSP1-ybbR sample likely represents separation of the dye-labeled (upper band) from the non-dye-labeled protein (lower band) in the 12% acrylamide gel.

C). Representative fluorescence image of a gel that depicts NSP1-ybbR-Cy5 association with purified and non-labeled human 40S ribosomal subunits at the indicated concentrations, as determined using native gel electrophoresis. NSP1-ybbR-Cy5 was present at 40 nM (by Cy5). Samples were incubated at 37 ºC for 10 minutes prior to native gel electrophoresis at 4 ºC. In the native gel assay and running buffer (pH 7.3), monomeric NSP1-ybbR (pI = 5.3, 21 kD) runs off the gel prior to entry and resolution of the ribosomal complexes. *n* = 3.

D). Representative fluorescence image of a gel that depicts NSP1-ybbR-Cy5 association with the indicated purified ribosomal subunits, as determined using native gel electrophoresis. 40 nM of NSP1-ybbR-Cy5 was incubated with 128 nM of the indicated subunits at 37 ºC for 10 minutes prior to native gel electrophoresis analysis at 4 ºC. In panels D and E, the identity of the band labeled as 'NSP1-NSP1' was assigned due to its appearance when labeled NSP1-ybbR was added alone and its increased intensity when excess unlabeled NSP1 was included in the assay. *n* = 3.

E). Representative fluorescence image of a gel that depicts NSP1-ybbR-Cy5 association with purified human 40S ribosomal subunits in the presence of the indicated competitor proteins present at 150-fold molar excess, as determined using native gel electrophoresis at 4 ºC. While unlabeled NSP1(WT) and NSP1(RK/AA) eliminated the 40S-NSP1-Cy5 band, substitution of KH to AA in unlabeled NSP1 yielded a strong 40S-NSP1-Cy5 band, similar to experiments in the absence of competitor protein. Thus, NSP1(KH/AA) was unable to associate stably with the human 40S subunit, as expected based on NSP1 from SARS-CoV-1. *n* = 3.

F). Representative fluorescence image of a gel that depicts NSP1-ybbR-Cy5 association with 128 nM purified human 40S ribosomal subunits in the presence of the indicated competitor proteins at 150-fold molar excess, as determined using native gel electrophoresis at 4 ºC. All lanes contained 40 nM NSP1-ybbR-Cy5. The identity of the NSP1–eIF1A–40S subunit band was confirmed using fluorescently-labeled 40S ribosomal subunits (panel D). The identity of the band labeled as 'NSP1-NSP1' was assigned due to its appearance when labeled NSP1-ybbR was added alone and its increased intensity when excess unlabeled NSP1 was included in the assay (also see SI Appendix, Fig. S2). *n* = 3.

G). Representative fluorescence image of a gel that depicts eIF1 enhanced NSP1 association with the human 40S ribosomal subunit, as determined using native gel electrophoresis at 4 ºC. All lanes contained 40 nM NSP1-ybbR-Cy5. *n* = 3.

H). Plot of the mean integrated densities of the indicated bands from the experiment depicted in panel B. At all examined concentrations, the presence of eIF1 increased the intensity of the 40S-NSP1-Cy5 band about 2-fold (mean fold increase = 2 ± 0.4). Error bars represent standard deviation of the mean. See **SI Appendix, Table S1** for raw data, calculated means, and standard deviations. *n* = 3.

I). Representative fluorescence image of a gel that depicts increased migration of the eIF1A–40S subunit complex in composite native gels. All lanes contained 40 nM NSP1-ybbR-Cy5 (red). 40S subunits were labeled with Cy3 (green) via uS19-ybbR (see, Figure 4 and SI Appendix, Fig. S6). The image is an overlay of the Cy3 and Cy5 fluorescence signals. *n* = 3.

Supplementary Figure 3

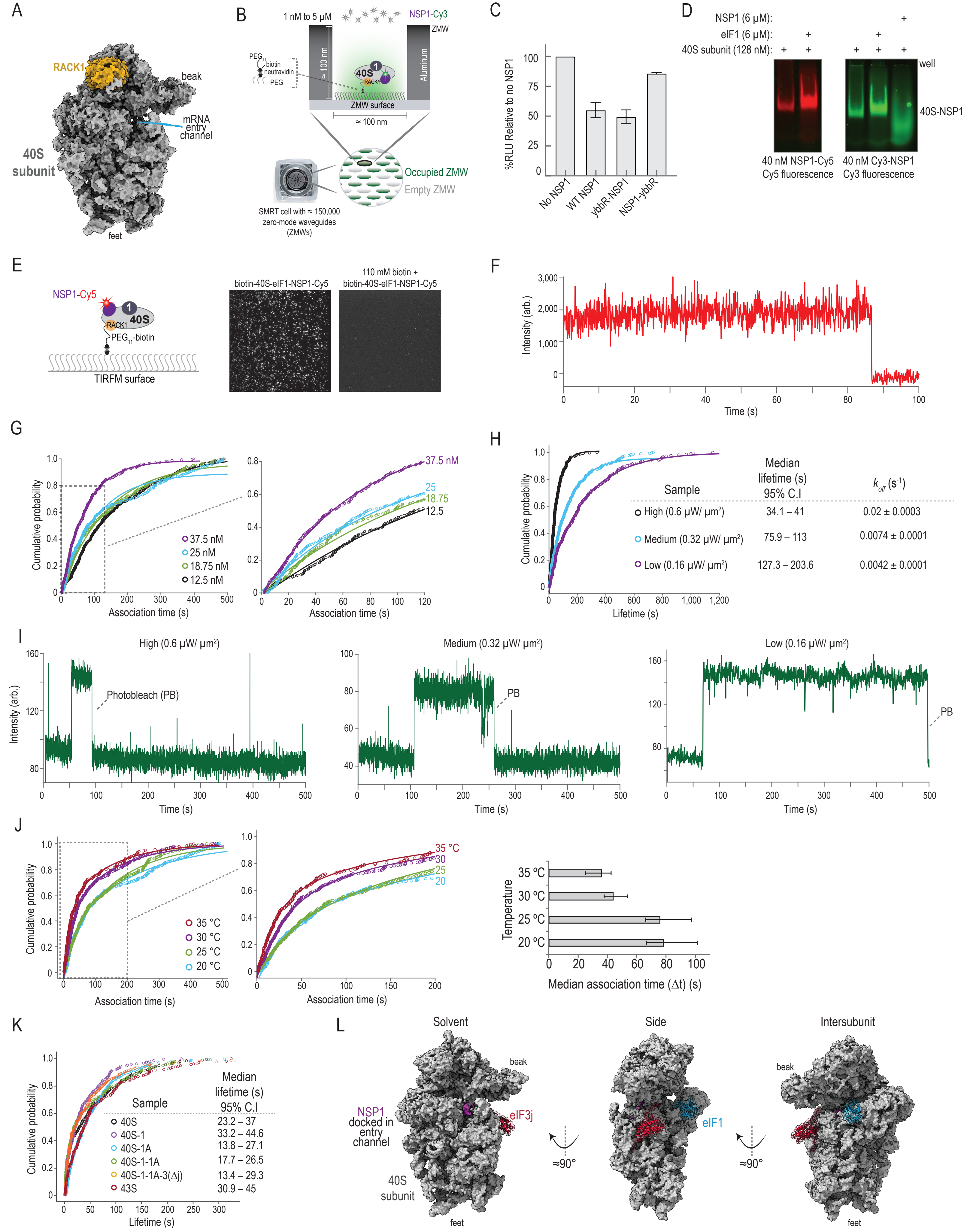

Figure S3. NSP1 bound 40S subunits within pre-initiation complexes. Related to **Fig. 2.**

A). Model of the human 40S ribosomal subunit (PDB: 5A2Q). The 40S subunit is depicted in gray and the ribosomal protein RACK1 is in purple.

B). Schematic of the zero-mode-waveguide (ZMW) imaging setup. One PacBio SMRT cell contains approximately 150,000 individual ZMWs, which enable direct monitoring of up to four fluorescent signals at concentrations up to 5 µM. The instrument (a PacBio RSII DNA sequencer) enables control of both experimental temperature and the power of the excitation lasers. In this scenario, purified 40S ribosomal subunits that contained RACK1 with a C-terminal ybbR-tag were purified from human cells. In the presence of Sfp synthase, PEG_{11}-biotin conjugated to co-enzyme A (co-A) was site-specifically attached to the first serine residue in the 11 amino acid ybbR tag (D**S**LEFIASKLA) on RACK1. Biotinylated 40S subunits (biotin-40S) were incubated with 30-fold molar excess eIF1 at 37 ºC for 15 minutes. eIF1–40S subunit complexes were tethered to the neutravidin-coated ZMW surface via the high-affinity biotin–neutravidin interaction. After washing away non-tethered components, data acquisition began at 20 ºC and Cy3-NSP1 was added. In this cartoon, ZMWs that contained a Cy3-NSP1 binding event during the experimental time frame are green, and ZMWs that lacked a binding event are gray.

C). Translation inhibition activity of the labeled proteins. Plot of mean nLuc signal from IVT reactions programed with GAPDH reporter mRNA in the absence of NSP1 or the presence of 0.4 µM of WT, N-terminally (ybbR-NSP1), or C-terminally tagged (NSP1-ybbR) NSP1. Error bars represent SEM. $n = 3.$

D). Representative fluorescence image of a gel that compares N-terminally (ybbR-NSP1) and C-terminally tagged (NSP1-ybbR) NSP1 association with purified human 40S ribosomal subunits. The Cy5 (left) and Cy3 (right) images were run in parallel on the same gel, but they were separated here for clarity. Wild-type, unlabeled NSP1 or eIF1 was added at 6 µM. 40 nM of labeled NSP1 (either Cy5-labeled or Cy3-labeled) was present. $n = 3$.

E). Schematic of total internal reflection fluorescence microscopy (TIRFM) experiments performed at equilibrium. Biotinylated 40S (biotin-40S) subunits were incubated with 12-fold molar excess of NSP1-Cy5 and 150-fold excess eIF1 at 37 ºC for 15 minutes. Pre-formed complexes were incubated on a TIRFM surface coated with neutravidin. After washing away unbound complexes and components, we imaged tethered ribosomal complexes at room temperature in the presence of 1 µM eIF1 and 4 nM of NSP1-Cy5. We observed a few hundred NSP1-Cy5 molecules tethered to the imaging surface in all view fields examined (representative view field shown, $n = 18$). Consistent with specific tethering, NSP1-Cy5 signal was eliminated by pre-saturation of the imaging surface with 110 mM biotin.

F). Example single-molecule fluorescence trace from the left view field of the equilibrium TRIFM experiment outlined in panel E (no biotin pre-treatment). The trace begins with fluorescence signal from NSP1-Cy5 (red) bound to a tethered eIF1–40S subunit complex.

G). Plot of the cumulative probability of Cy3-NSP1 association times at the indicated concentrations with tethered eIF1–40S subunit complexes at 20 ºC. The left plot depicts all association times, and the right plot highlights the subset within the dashed box of the left plot. Lines represent fits to single-exponential functions. See **SI Appendix, Table S2** for samples sizes and the parameters for relevant fits.

H). Plot of the cumulative probability of Cy3-NSP1 lifetimes on eIF1–40S subunit complexes at 20 ºC and the indicated excitation laser powers. Cy3-NSP1 was added at 25 nM. The wavelength of the excitation laser was 532 nm, and it was used at high (0.6 µW/ μm^2), medium (0.32 µW/ μm^2), and low (0.16 µW/ μm^2) power to determine whether the lifetime of the NSP1 fluorescence signal was limited by the photostability of the Cy3 dye. Lines represent (left plot) fits to exponential functions, which were used to derive k_{off}. See panel I for example traces and **SI Appendix, Table S2** for samples sizes and the parameters for relevant fits.

I). Example single-molecule fluorescence trace that depicts association of Cy3-NSP1 (green) with a tethered eIF1–40S subunit complex acquired at different laser powers. The wavelength of the excitation laser was 532 nm, and it was used at high (0.6 µW/ μm^2), medium (0.32 µW/ μm^2), and low (0.16 µW/ μm^2) power to determine whether the lifetime of the Cy3-NSP1 fluorescence signal was limited by the photostability of the Cy3 dye. During imaging, eIF1 was present at 1 µM. Loss of fluorescence due to likely photobleaching of the Cy3 dye is indicated on each trace.

J). Plot of the cumulative probability of Cy3-NSP1 association times with tethered eIF1–40S subunit complexes at the indicated temperatures. In all experiments, Cy3-NSP1 was present at 25 nM. The left plot depicts all association times, and the middle plot highlights the subset within the dashed box of the left plot. Lines represent fits to double-exponential functions. The bar chart (right) depicts median association times at the indicated temperatures. Error bars represent 95% C.I.. See **SI Appendix, Table S2** for samples sizes and the parameters for relevant fits.

K). Plot of the cumulative probability of Cy3-NSP1 lifetimes on the indicated 40S pre-initiation complexes. In all experiments, Cy3-NSP1 was present at 25 nM and the temperature was 30 ºC. See **SI Appendix, Table S2** for samples sizes and medians with 95% C.I..

L). Model of the human 40S ribosomal subunit bound by eIF1 (blue), eIF3j (red), and NSP1 (purple). The 40S subunit, eIF1, and eIF3j were modelled using ChimeraX and the PDB: 6ZMW. Using the mmaker command, the NSP1-40S subunit complex (PDB: 6ZLW) was aligned to the ribosomal pre-initiation complex to model NSP1 docked into the mRNA entry channel of the complex.

Supplementary Figure 4

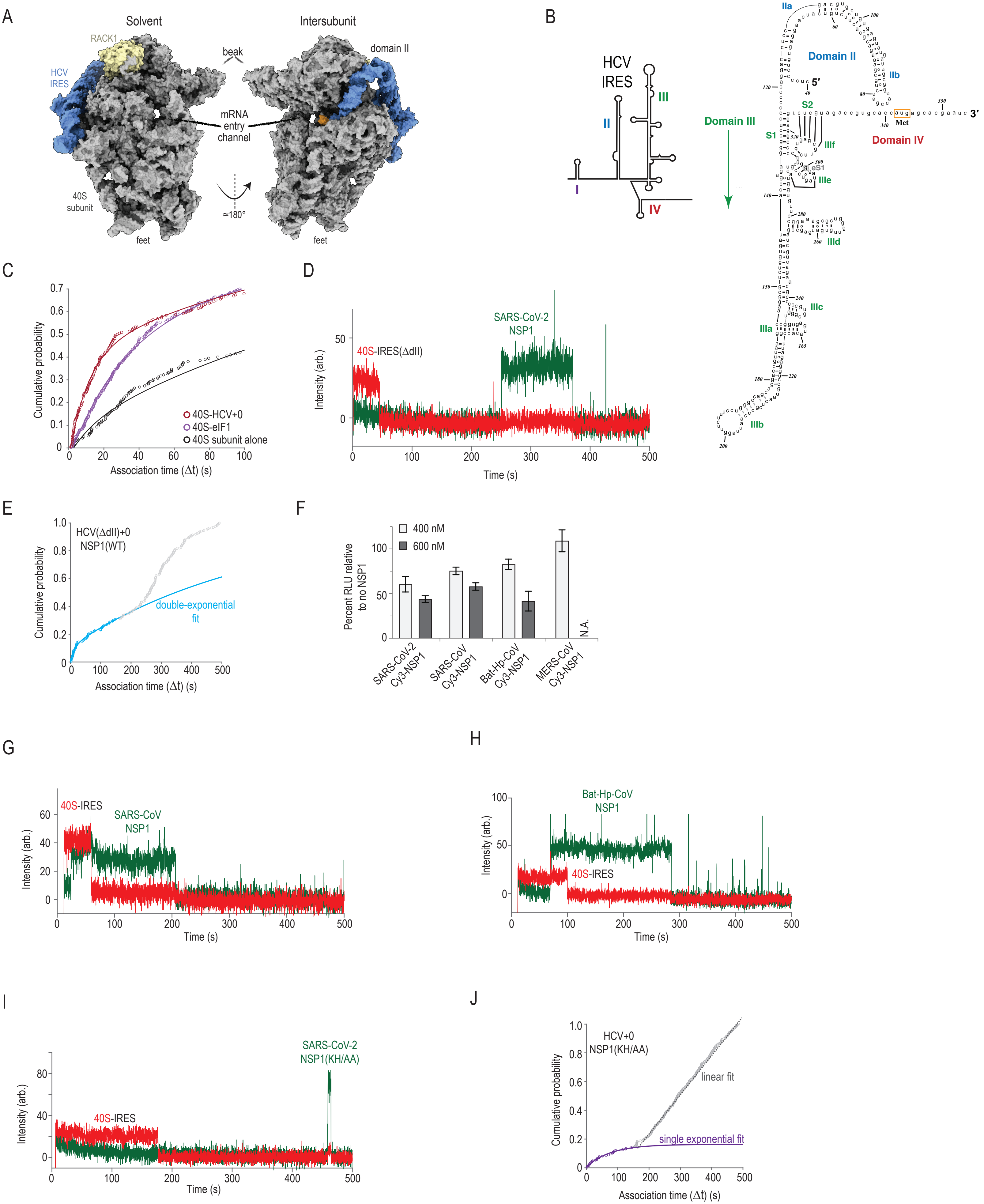

Figure S4. mRNA within the mRNA entry channel of the 40S subunit inhibited NSP1 association. Related to **Fig. 3.**

A). Model of the human 40S subunit bound by the HCV IRES (PDB: 5A2Q). The 40S subunit is depicted in gray, RACK1 in light yellow, and the HCV IRES in blue. The model of the IRES ends at the start codon (AUG, highlighted in orange), leaving the mRNA entry channel of the 40S subunit empty. Domain II of the IRES holds the head of the 40S subunit in the open conformation.

B). Secondary structure of the HCV IRES (adapted from Johnson et al., ref. 50).

C). Plot of the cumulative probability of Cy3-NSP1 association times with 40S subunit complexes. In all experiments, Cy3-NSP1 was present at 25 nM and the temperature was 30 ºC. The data for the 40S subunit alone (40S) and the eIF1–40S subunit complex (40S–eIF1) were replotted here from **Figs. 2E & 3E** for comparison to the data for NSP1 association with the 40S–HCV+0 complex. Lines represent fits to single- or double-exponential functions. See **SI Appendix, Table S3** for samples sizes and the parameters for relevant fits.

D). Example single-molecule fluorescence trace that depicts a tethered 40S-HCV(ΔdII)+0 complex and subsequent association of SARS-CoV-2 NSP1. The 40S subunit and ybbR-NSP1 were labeled with Cy5 (red) and Cy3 (green) dyes, respectively. Raw fluorescence intensities were corrected in this image to set baseline intensities to zero for presentation.

E). Cumulative probability plot of SARS-CoV-2 NSP1 association times (Δt) at 25 nM at 30 ºC. Given the multiphasic behavior, we fit the events that occurred within the first 150 s to a single-exponential function (purple circles and line) to estimate the fast association rate. See **SI Appendix, Table S3** for samples sizes and the parameters for relevant fits.

F). Translation inhibition activity of the indicated fluorescently-labeled proteins. Plot of mean nLuc signal from IVT reactions programed with GAPDH reporter mRNA in the presence of 0.4 or 0.6 μM N-terminally tagged Cy3-NSP1 from SARS-CoV-2, SARS-CoV, Bat-Hp-CoV, or MERS-CoV. N.A., experiment not done due to low concentration of the labeled protein. Error bars represent SEM. Bat-Hp-CoV*,* *n = 4,* all others *n = 3).*

G, H, I). Example single-molecule fluorescence traces that depict a tethered 40S-HCV+0 complex and subsequent association of SARS-CoV NSP1 (**G**), Bat-Hp-CoV NSP1 (**H**), and SARS-CoV-2 NSP1(KH/AA) (**I**) proteins. The 40S subunit and ybbR-NSP1 proteins were labeled with Cy5 (red) and Cy3 (green) dyes, respectively. Raw fluorescence intensities were corrected in the images to set baseline intensities to zero for presentation.

J). Cumulative probability plot of SARS-CoV-2 NSP1(KH/AA) association times (Δt) at 25 nM at 30 ºC. Given the multiphasic behavior, we fit the events that occurred within the first 150 s to a double-exponential function (blue circles and line) to estimate the fast association rate. See **SI Appendix, Table S3** for samples sizes and the parameters for relevant fits.

Supplementary Figure 5

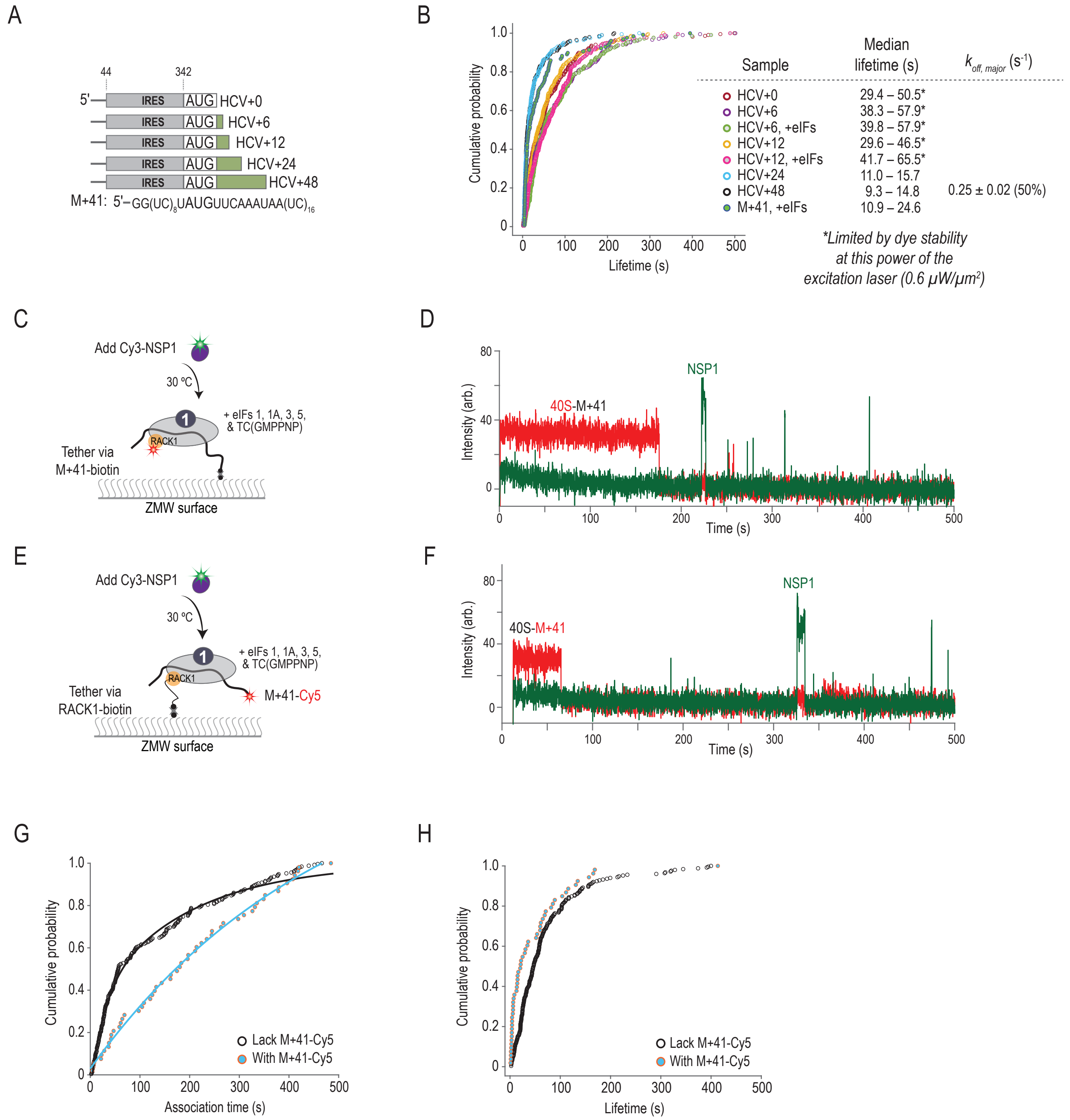

Figure S5. mRNA within the entry channel of the 40S subunit inhibited SARS-CoV-2 NSP1 association. Related to **Fig. 4.**

A). Schematic of model mRNAs. HCV IRES RNAs were biotinylated on the 5'-terminus and contained 0, 6, 12, 24, or 48 nucleotides downstream of the start codon (AUG). All HCV IRES RNAs used contained domain I of the HCV genomic RNA. The model mRNA (M+41) contained 41 nucleotides downstream of the start codon, and it was biotinylated or labeled with Cy5 on the 3'-terminus.

B). Plot of the cumulative probability of observed SARS-CoV-2 Cy3-NSP1 lifetimes with the indicated mRNA–40S subunit complexes at 30 ºC. Cy3-NSP1 was added at 25 nM (final concentration) in all experiments. '+eIFs' indicates eIFs 1, 1A, 3, 5, and TC(GMPPNP) were included at all stages of the experiment. The excitation laser (532 nm) for NSP1 was at 0.6 $\mu W/\mu m^2$ for these experiments, which resulted in artificially shortened lifetimes for the indicated interactions. Lines represent fits to double-exponential functions. See **SI Appendix, Table S4** for samples sizes and the parameters for relevant fits.

C). Schematic of the single-molecule fluorescence experimental setup with biotinylated M+41 model mRNA. 40S ribosomal subunits were labeled with Cy5 dye via RACK1-ybbR. M+41 RNA was labeled on the 3'-terminus with biotin. Pre-formed M+41–40S-Cy5 complexes were tethered to the ZMW imaging surface in the presence of eIF1, eIF1A, eIF3, eIF5, and TC-GMPPNP. At the start of data acquisition, Cy3-NSP1 was added at 25 nM at 30 °C.

D). Example single-molecule fluorescence trace that depicts a tethered M+41–40S-Cy5 complex and subsequent association of Cy3-NSP1. The 40S subunit and ybbR-NSP1 were labeled with Cy5 (red) and Cy3 (green) dyes, respectively. Raw fluorescence intensities were corrected in this image to set baseline intensities to zero for presentation. The association time (Δt) was defined as time elapsed from the addition of Cy3-NSP1 until the burst of Cy3 fluorescence (green), which signified NSP1 association. The lifetime was defined as the duration of the Cy3 fluorescence signal.

E). Schematic of the single-molecule fluorescence experimental setup with fluorescently-labeled M+41 model mRNA. 40S ribosomal subunits were labeled with biotin via RACK1-ybbR. M+41 RNA was labeled on the 3'-terminus with Cy5. Pre-formed Cy5-M+41–40S subunit complexes were tethered to the ZMW imaging surface in the presence of eIF1, eIF1A, eIF3, eIF5, and TC-GMPPNP. At the start of data acquisition, Cy3-NSP1 was added at 25 nM at 30 °C.

F). Example single-molecule fluorescence trace that depicts a tethered Cy5-M+41–40S subunit complex and subsequent association of Cy3-NSP1. The M+41 RNA and ybbR-NSP1 were labeled with Cy5 (red) and Cy3 (green) dyes, respectively. Raw fluorescence intensities were corrected in this image to set baseline intensities to zero for presentation. The association time (Δt) was defined as time elapsed from the addition of Cy3-NSP1 until the burst of Cy3 fluorescence (green), which signified NSP1 association. The lifetime was defined as the duration of the Cy3 fluorescence signal.

G,H). Cumulative probability plots of the observed association times (panel G) and lifetimes (panel H) of Cy3-NSP1 with the indicated 40S–mRNA complexes. Biotinylated 40S subunits (as in **Fig. 2** & **SI Appendix. Fig. S4**) were directly tethered to the surface in the presence of the model mRNA (M+41) labeled on the 3'-terminus with Cy5 and eIFs 1,1A, 5, and TC-GMPPNP. Both sets of molecules plotted were here were obtained from the same experiment. The 'Lack M+41' data represent ZMWs that had at least one Cy3-NSP1 binding event but lacked Cy5-M+41 signal. The 'With M+41' data represent ZMWs that began with Cy5 signal (Cy5-M+41) and contained at least one NSP1 binding event (Cy3) longer than $\approx$ 5s. Cy3-NSP1 was present at 25 nM and the temperature was 30 ºC. See **SI Appendix, Table S4** for samples sizes, parameters for relevant fits, and medians with 95% C.I..

Supplementary Figure 6

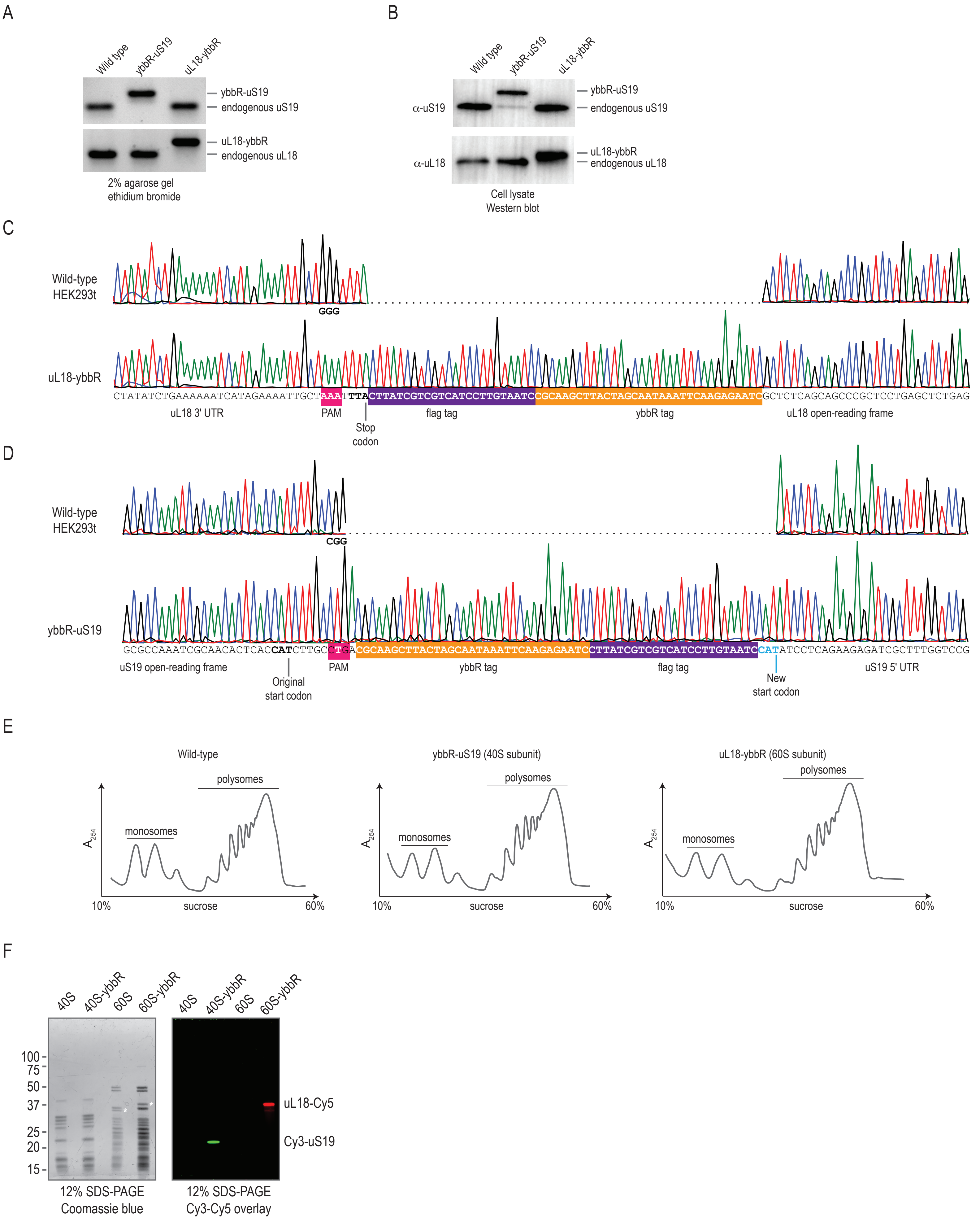

Figure S6. Generation of an inter-subunit FRET signal for the human ribosome. Related to **Fig. 5**.

A). Image of a 2% agarose gel stained with ethidium bromide. The analyzed PCR products were generated with primers that flanked the desired insertion site for the ybbR-flag tag, with at least one of them lying outside the repair template.

B). Image of Western blots using the indicated antibodies on whole cell extract from the indicated cell lines. Together with the PCR data from panel B, these data indicated that all endogenous copies of uS19 or uL18 were edited to incorporate ybbR-flag tags. The faint band that may represent endogenous uS19 in the ybbR-uS19 sample of the Western blot could result from translation initiation at the original start codon, rather than the new, inserted start codon.

C, D). Traces from Sanger sequencing of PCR products generated as in panel B for the indicated cell lines and genomic loci. Important regions within the sequenced DNA segments are annotated. For both ybbR-uS19 and uL18-ybbR cell lines, the ybbR-flag tag was inserted as designed and the used PAM sites were disrupted, as encoded in the repair templates.

E). Representative traces from polysome profiling assays on the indicated cell lines. The positions of monosomes (individual ribosomal subunits and single 80S ribosomes) and polysomes (multiple 80S ribosomes) are indicated. Similar levels of polysomes were detected in wild-type, ybbR-uS19, and uL18-ybbR HEK293t cell lines. Thus, ribosomes that contain the ybbR-flag tag on uS19 or uL18 were functional. $n = 2$.

F). Gel that depicts purified and fluorescently-labeled ribosomal subunits. Ribosomal subunits were purified from either the HEK293T cell line that expressed ybbR-uS19 (40S-ybbR & 60S samples) or uL18-ybbR (40S & 60S-ybbR samples). Following ybbR-labeling reactions with either Cy3 (green) or Cy5 (red) dyes, ribosomal subunits were analyzed by SDS-PAGE on 12% acrylamide gels. Prior to staining total protein with Coomassie blue (left image), gels were scanned for both Cy3 and Cy5 fluorescence (right image), which is presented as an overlay of both fluorescent signals.

Supplementary Figure 7

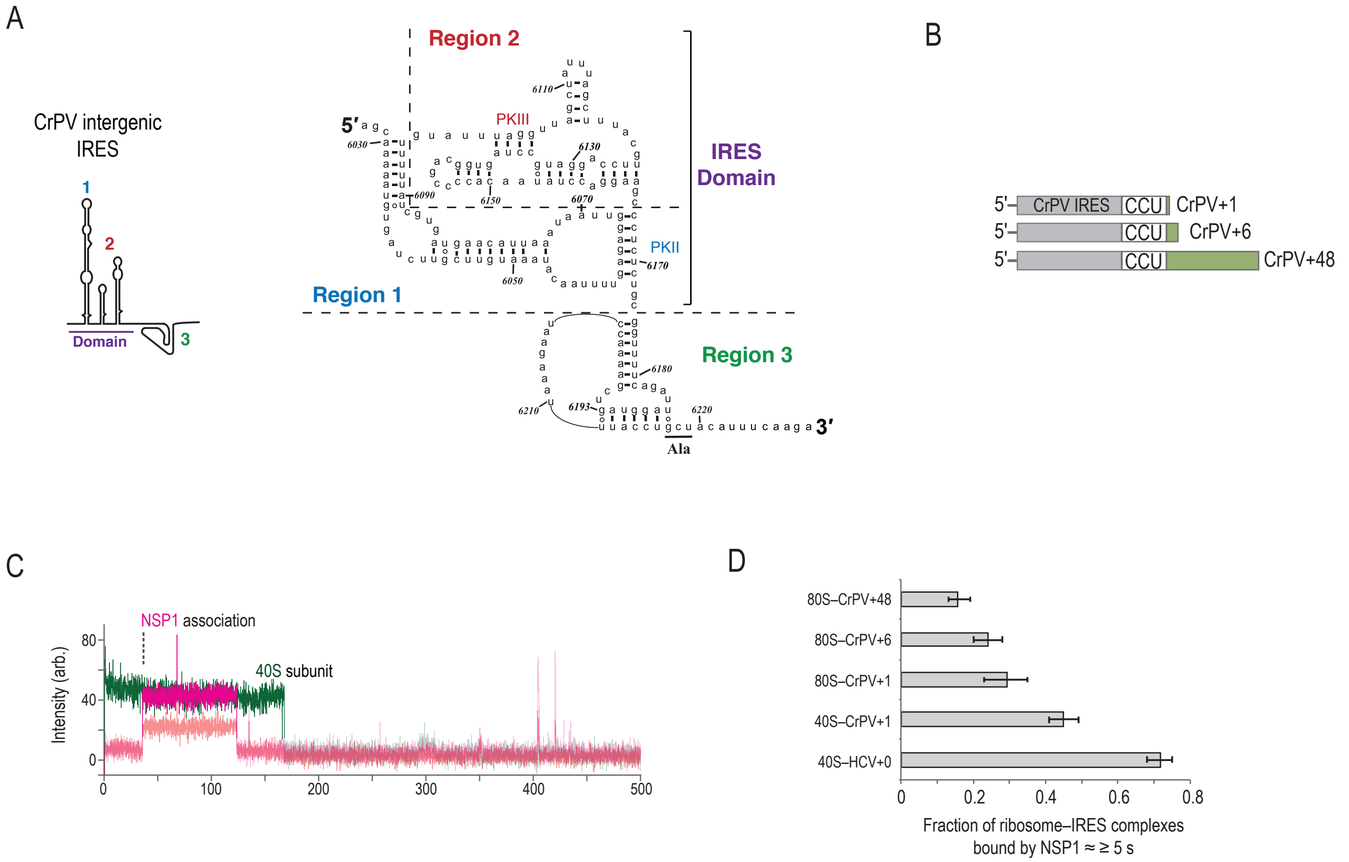

Figure S7. NSP1 inefficiently bound to 80S–CrPV IRES complexes. Related to **Fig. 5**.

A). Secondary structure of the cricket paralysis virus (CrPV) intergenic (IGR) IRES (adapted from Johnson et al., ref. 50). All CrPV IRES models contained region 3 and had the first codon (Ala) mutated to a Phe codon (TTC).

B). Schematic of CrPV IRES RNAs used in the assays. The RNAs contained 1, 6, or 48 nucleotides downstream of the CCU codon in the CrPV IRES, which is present in the ribosomal A site. All RNAs were biotinylated on the 5'-terminus.

C). Example single-molecule fluorescence trace that depicts addition of Cy5.5-NSP1 to tethered 40S–CrPV+1 complexes. The 40S subunit was labeled with Cy3 (green) and NSP1 with Cy5.5 (magenta). Due to bleed through across the three fluorescent channels, the Cy3, Cy5, and Cy5.5 signals were made transparent before and after relevant events for presentation here. The association time (Δt) was defined as the time elapsed from the addition of Cy5.5-NSP1 until the burst of Cy5.5 fluorescence (magenta), which signified NSP1 association. The lifetime was defined as the duration of the Cy5.5 fluorescence signal.

D). Plot of the fraction of the indicated ribosome–IRES complexes bound at least once by NSP1 for $\geq \approx 5$s. Error bars represent 95% C.I. yielded from bootstrapping analyses.

Supplementary Figure 8

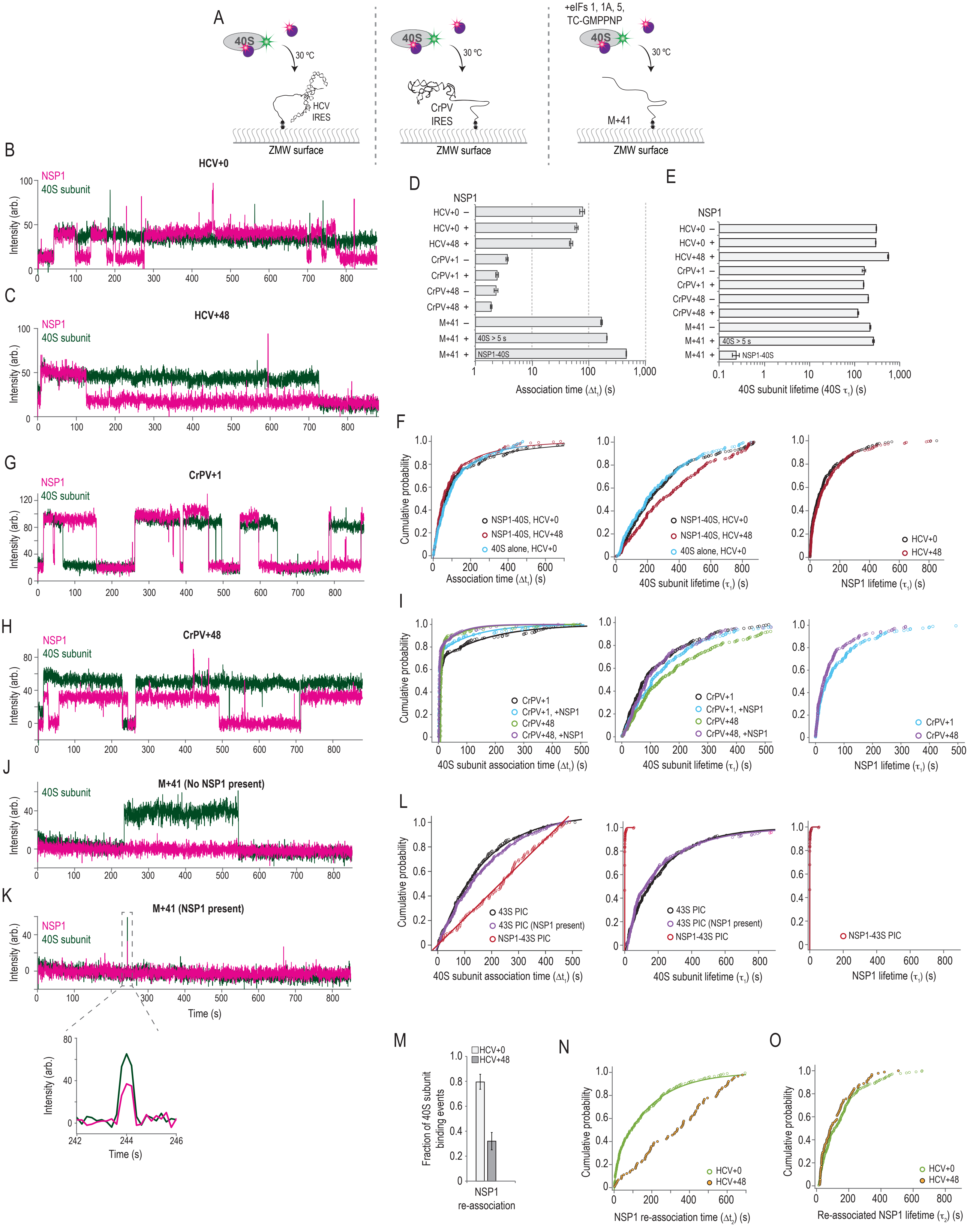

Figure S8. NSP1 remained bound to 40S subunits upon association with model mRNAs. Related to **Fig. 6**.

A). Schematic of the three single-molecule fluorescence experiments performed with the HCV IRES, CrPV IRES, and M+41 model mRNAs. 40S ribosomal subunits were labeled with Cy3 dye via uS19-ybbR. When indicated, NSP1–40S subunit complexes were pre-formed by incubating 2-fold molar excess Cy5.5-NSP1 with 40S-Cy3 subunits at 37 °C for 15 minutes. After tethering of model RNAs in ZMWs and start of data acquisition, a final concentration of 15 nM (by 40S subunits) 40S-Cy3 subunits or NSP1–40S subunit complexes were added at 30 °C. For experiments with M+41, eIFs 1, 1A, 5, and TC-GMPPNP were present during formation of the NSP1–40S subunit complex and during data collection to promote formation of the NSP1–43S PIC.

B,C). Example single-molecule fluorescence traces that depict association of NSP1–40S subunit complexes with tethered HCV+0 (panel B) or HCV+48 (panel C) IRES molecules. The 40S subunit and NSP1 were labeled with Cy3 (green) and Cy5.5 (magenta) dyes, respectively. Raw fluorescence intensities were corrected in this image to set baseline intensities to zero for presentation. These traces depict the full duration of data acquisition. In both, a single NSP1–40S subunit complex associates with the HCV IRES. Following initial loss of NSP1 signal, NSP1 readily re-associated with the same 40S–HCV+0 complex but not the 40S–HCV+48 complex.

D,E). Plot of initial association times (panel D) and 40S subunit lifetimes (panel E) from the indicated experiments. The presence or absence of NSP1 is indicated. The initial association time (Δt_1) was defined as the reciprocal of the fast association rate derived from fits to double-exponential functions. The 40S subunit lifetime (40S $\tau 1$) was defined as the reciprocal of the predominate dissociation rate derived from fits to double-exponential functions. In both, error bars represent 95% C.I. of the rate. When NSP1-43S PIC complexes were added to M+41 mRNA, we examined the lifetime of stable 40S subunit binding events (40S > 5s), which almost always lacked NSP1 signal, and the lifetime of co-recruited NSP1-43S PIC complexes (NSP1-40S). See **SI Appendix, Table S6** for samples sizes and the parameters for relevant fits.

F). Cumulative probability plot of observed initial association times (Δt_1, left plot), 40S subunit lifetimes (40S $\tau 1$, middle plot), and initial NSP1 lifetimes (NSP1 $\tau 1$, right plot) of either 40S subunits alone or NSP1–40S subunit complexes with HCV+0 and HCV+48 model RNAs at 30 ºC. Lines represent fits to double-exponential functions. See **SI Appendix, Table S6** for samples sizes and the parameters for relevant fits.

G,H). Example single-molecule fluorescence traces that depict association of NSP1–40S subunit complexes with a tethered CrPV+1 (panel G) or CrPV+48 (panel H) IRES molecules. The 40S subunit and NSP1 were labeled with Cy3 (green) and Cy5.5 (magenta) dyes, respectively. Raw fluorescence intensities were corrected in this image to set baseline intensities to zero for presentation. These traces depict the full duration of data acquisition. The 40S–CrPV IRES complex was more transient than that of the 40S–HCV IRES complex. Thus, we often observed multiple NSP1–40S subunit complex association events within a single ZMW, particularly for CrPV+1, which prevented analysis of NSP1 re-association with these model RNAs.

I). Cumulative probability plot of observed initial association times (Δt_1, left plot), 40S subunit lifetimes (40S $\tau 1$, middle plot), and initial NSP1 lifetimes (NSP1 $\tau 1$, right plot) of either 40S subunits alone or NSP1–40S subunit complexes with CrPV+1 and CrPV+48 model RNAs at 30 ºC. Lines represent fits to double-exponential functions. See **SI Appendix, Table S6** for samples sizes and the parameters for relevant fits.

J,K). Example single-molecule fluorescence traces that depict association of 43S PIC (eIFs 1, 1A, 5, TC-GMPPNP) (panel J) or NSP1–43S PIC (panel K) with tethered M+41 model mRNAs. The 40S subunit and NSP1 were labeled with Cy3 (green) and Cy5.5 (magenta) dyes, respectively. eIFs 1, 1A, 5, and TC-GMPPNP were present during formation of the NSP1–40S subunit complex and during data collection to promote formation of the NSP1–43S PIC. Raw fluorescence intensities were corrected in this image to set baseline intensities to zero for presentation. The inset on panel K represents a zoomed-in view of the typical transient NSP1–40S subunit binding event.

L). Cumulative probability plot of observed initial association times (Δt_1, left plot), 40S subunit lifetimes (40S $\tau 1$, middle plot), and initial NSP1 lifetimes (NSP1 $\tau 1$, right plot) of either 40S subunits alone or NSP1–40S subunit complexes with the M+41 model RNAs at 30 ºC. Lines represent fits to double-exponential functions. See **SI Appendix, Table S6** for samples sizes and the parameters for relevant fits.

M). Plot of the fraction of 40S subunit binding events that had at least one NSP1 re-association event (right) with the HCV+0 or HCV+48 complexes. As noted in **Fig. 6B**, we focused on ZMWs where a single 40S subunit associated within the first 200 s ($\approx$ 75% of all events, see **SI Appendix, Fig. S8F**). We then quantified the time elapsed from the loss of the first Cy5.5 signal to the next burst of Cy5.5 fluorescence at least $\approx$ 20 s in length ($\approx$ 70% of initial NSP1 binding events, see **SI Appendix, Fig. S8F**), which was defined as the NSP1 re-association time (NSP1 Δt_2). The duration of this second Cy5.5 event was defined as the re-associated NSP1 lifetime (NSP1 τ_2)., as defined below in panel B. Error bars represent 99% C.I..

N,O). Cumulative probability plots of observed NSP1 re-association times (panel O) or lifetimes (panel P) with the indicated IRES–40S subunit complexes at 30 ºC. As outlined in **Fig. 4A**, we focused on ZMWs where a single 40S subunit associated within the first 200 s ($\approx$ 75% of all events, see panel D). We then quantified the time elapsed from the loss of the first Cy5.5 signal to the next burst of Cy5.5 fluorescence at least $\approx$ 20 s in length ($\approx$ 70% of initial NSP1 binding events, see panel F), which was defined as the NSP1 re-association time (NSP1 Δt_2). The duration of this second Cy5.5 event was defined as the re-associated NSP1 lifetime (NSP1 τ_2). Lines represent fits to double-exponential functions. See **SI Appendix, Table S6** for samples sizes, parameters for relevant fits, and medians with 95% C.I..

Supplementary Table 1. Related to Supplementary Figure 2G & H

Gel	Condition	NSP1 (nM)	Integrated Density	Mean Integrated Density	St. Dev	Fold change (eIF1/none)
Gel 1	None	32	395.5	400.2	10.8	
		64	879.1	838.7	48.1	
		90	1187.2	1047.6	122.7	
		128	1194.2	1211.7	37.3	
	6 µM eIF1	32	1043.3	1036.8	60.7	2.59
		64	1737.6	1667.4	139.4	1.99
		90	1964.9	1871.8	168.3	1.79
		128	2093.1	1990.3	170.3	1.64
Gel 2	None	32	392.6		**Mean Fold change:**	**2.00**
		64	851.4		**Standard deviation:**	**0.42**
		90	998.6			
		128	1254.5			
	6 µM eIF1	32	1094.1			
		64	1757.7			
		90	1973.0			
		128	2083.9			
Gel 3	None	32	412.6			
		64	785.5			
		90	957.0			
		128	1186.5			
	6 µM eIF1	32	973.1			
		64	1506.9			
		90	1677.5			
		128	1793.7			

Supplementary Table 2.

Related to Figure 2 and Supplementary Figure 3

Related to Figure 2C

	ZMWs with Cy3 binding event (non-sample)					
Tethered complex (20 °C)	NSP1 (nM)	eIF3j (µM)	>= 1 Cy3 event	Traces (n)	Fraction	99% C.I.
None	75		27	300	0.09	0.05 to 0.13
eIF1-40S subunits	75		168	300	0.56	0.49 to 0.63
eIF1-40S subunits	12.5		144	300	0.48	0.4 to 0.55
eIF1-40S subunits	12.5	6	17	300	0.06	0.03 to 0.09
eIF1-40S subunits	25		180	300	0.60	0.53 to 0.67
eIF1-40S subunits	25	6	22	300	0.07	0.04 to 0.12

Related to Figure 2D and Supp. Figure 3G

		Association rates and amplitudes from single-exponential fits					Median association time	
Tethered complex (20 °C)	NSP1 (nM)	Amplitude (Amp.)	Amp. error	Fast association rate (per s)	Rate error (95% C.I.)	n	Median (s)	95% CI (s)
eIF1-40S subunits	12.5	1.04	0.01	0.0058	0.0001	323	112.5	98.2 to 129.4
eIF1-40S subunits	18.75	0.97	0.01	0.0073	0.0003	309	93.6	75 to 115
eIF1-40S subunits	25	0.89	0.01	0.0095	0.0004	321	78.3	66.4 to 100.7
eIF1-40S subunits	37.5	0.99	0.004	0.0140	0.0002	316	51.4	43.5 to 64.7

Related to Supp. Figure 3H

			Lifetime			Lifetimes and amplitudes from fits to single-exponential functions				
532 nM laser power (µW/µm2)	Tethered complex (20 °C)	NSP1 (nM)	Median (s)	95% C.I. (s)	n	Amp. 1 (A1)	A1 error	Fast rate (k1, per s)	k1 error	adj. R2
0.6	eIF1-40S subunits	25	38.8	34.1 to 41	321	1.01	0.01	0.020	0.0003	0.997
0.32	eIF1-40S subunits	25	96.2	75.9 to 113	306	0.96	0.00	0.0074	0.0001	0.999
0.16	eIF1-40S subunits	25	168	127.3 to 203.6	213	0.99	0.01	0.0042	0.0001	0.996

Related to Supp. Figure 3J

			Median association time			Association rates and amplitudes from double-exponential fits							
Tethered complex	Temperature (°C)	NSP1 (nM)	Median (s)	95% CI (s)	n	Amp. 1 (A1)	A1 error	Fast rate (k1, per s)	k1 error	Amp. 2 (A2)	Slow rate (k2, per s)	k2 error	adj. R2
eIF1-40S subunits	20	25	78.3	66.8 to 101.4	321	0.32	0.023	0.032	0.004	0.68	0.0047	0.0002	0.996
eIF1-40S subunits	25	25	75.9	66.4 to 97.4	308	0.19	0.028	0.038	0.008	0.81	0.0063	0.0002	0.995
eIF1-40S subunits	30	25	43.95	38 to 53.7	316	0.47	0.029	0.035	0.002	0.53	0.0065	0.0003	0.998
eIF1-40S subunits	35	25	36.05	25.2 to 42.4	202	0.51	0.021	0.049	0.003	0.49	0.0072	0.0003	0.998

Related to Figure 2E,F and Supp. Figure 3K

		Association time		Lifetime			Association rates and amplitudes from exponential fits								Estimated lifetimes from the predominate phase yielded				
Tethered complex (30 °C)	NSP1 (nM)	Median (s)	95% CI (s)	Median (s)	95% CI (s)	n	Amp. 1 (A1)	A1 error	Fast rate (k1, per s)	k1 error	Amp. 2 (A2)	Slow rate (k2, per s)	k2 error	adj. R2	Amplitude	Amp. Error	Rate (k1, per s)	k1 error	adj. R2
40S	25	112.5	91 to 136.8	30.6	23.2 to 37	205	0.10	0.03	0.075	0.07	0.90	0.005	0.0002	0.976	0.73	0.01	0.014	0.0002	0.999
40S-1	25	44.0	38 to 53.7	38.0	33.2 to 44.6	316	0.47	0.03	0.035	0.002	0.53	0.007	0.0003	0.998	0.93	0.01	0.018	0.0002	0.999
40S-1A	25	89.8	53.4 to 131.1	18.5	13.8 to 27.1	204	0.25	0.02	0.067	0.01	0.75	0.005	0.0002	0.990	0.62	0.02	0.012	0.0004	0.997
40S-1-1A	25	49.6	35.1 to 63.4	21.4	17.7 to 26.5	217	0.39	0.01	0.052	0.003	0.61	0.006	0.0002	0.998	0.81	0.01	0.023	0.0004	0.998
40S-1-1A-3	25	74.5	61.8 to 103.2	20.7	13.4 to 29.3	211	0.29	0.03	0.049	0.01	0.71	0.005	0.0003	0.991	0.68	0.01	0.017	0.0001	0.999
40S-1-1A-3-TC-5	25	60.4	47.1 to 86.4	38.2	30.9 to 45	217	0.42	0.02	0.034	0.002	0.58	0.004	0.0001	0.998	0.56	0.1	0.010	0.0001	0.997

Supplementary Table 3. Related to Figure 3 and Supplementary Figure 4

Related to Figure 3D

Tethered complex (30 °C)	NSP1 (nM)	NSP1 Identity	Traces (n)	>= 1 Cy3 event	Fraction	+/- 99% C.I.
40S-HCV +0 (rep. 1)	25	SARS-CoV-2, WT	1005	778	0.77	0.03
40S-HCV(ΔII)+0	25	SARS-CoV-2, WT	1004	600	0.60	0.04
40S-HCV+0	25	SARS-CoV-2, KH/AA mutant	1006	87	0.09	0.02
40S-HCV+0	25	SARS-CoV, WT	1004	622	0.62	0.04
40S-HCV+0	25	Bat-Hp Zhejiang, WT	1009	893	0.89	0.02
40S-HCV+0	25	MERS-CoV, WT	1004	112	0.11	0.02

Related to Figure 3E,G and Supp. Fig. 4E,J

			Association rates and amplitudes from double-exponential fits										Median association time			
Tethered complex (30 °C)	NSP1 (nM)	NSP1 Identity	Amp. 1 (A1)	A1 error	Fast rate (k1, per s)	k1 error	Amp. 2 (A2)	Slow rate (k2, per s)	k2 error	adj. R2	n	Type of fit	Median (s)	95% CI (s)	1/Kon	error
40S-HCV +0 (rep. 1)	25	SARS-CoV-2, WT	0.41	0.01	0.095	0.006	0.59	0.0053	0.0001	0.998	217	DE	36.3	24.5 to 58.8	10.5	0.6
40S-HCV(ΔII)+0	25	SARS-CoV-2, WT	0.11	0.01	0.07	0.010	NA	NA	NA	NA	222	SE*	260.9	241.9 to 276.6	14.3	1.8
40S-HCV+0	25	SARS-CoV-2, KH/AA mutant	0.16	0.01	0.010	0.003	NA	NA	NA	0.99	245	SE**	292.2	276.4 to 316	100.0	20.1
			NA	NA	0.0027	0.00002				0.997		Linear estimate***			NA	NA
40S-HCV+0	25	SARS-CoV, WT	0.15	0.02	0.096	0.040	0.85	0.0060	0.0006	0.987	216	DE	109.6	71.3 to 137.7	10.4	3.1
40S-HCV+0	25	Bat-Hp Zhejiang, WT	0.64	0.02	0.060	0.003	0.36	0.0063	0.0004	0.997	222	DE	21.7	17.7 to 29.8	16.6	0.8

**restricted to events that ocurred within 100 s to estimate fast rate
**restricted to events that ocurred within 150 s to estimate fast rate
***events that occurred after 150 s
DE = double exponential
SE = single exponential

Related to Figure 3F,G

				Lifetime			Estimated lifetimes from the predominate phase yielded by fits to double-exponential functions							
Tethered complex (30 °C)	NSP1 (nM)	NSP1 Identity	Excitiation laser power (532 nM) μW/μm2	Median (s)	95% CI (s)	n	Amplitude	Amp. Error	Rate (k1, per s)	k1 error	adj. R2	Reported in Fig. 2F,G	1/Koff	error
40S-HCV +0 (rep. 1)	25	SARS-CoV-2, WT	0.6	47.2	40.2-57.1	217	0.80	0.07	0.013	0.001	0.999	No		
40S-HCV(ΔII)+0	25	SARS-CoV-2, WT	0.6	42.7	37.7-54.6	222	0.84	0.01	0.013	0.0006	0.998	No		
40S-HCV+0	25	SARS-CoV-2, KH/AA mutant	0.6	12.1	10.4-14.9	245	0.66	0.02	0.14	0.008	0.997	**Yes**	7.1	0.4
40S-HCV+0	25	SARS-CoV, WT	0.6	38.9	36.3-47.6	216	0.70	0.15	0.026	0.004	0.996	No		
40S-HCV+0	25	Bat-Hp Zhejiang, WT	0.6	50.6	38.9-58.8	222	0.58	0.03	0.007	0.0004	0.998	No		
40S-HCV+0	25	SARS-CoV-2, WT	0.1	100.0	79-131	213	0.74	0.02	0.0043	0.0001	0.999	**Yes**	232.6	5.3
40S-HCV+0	25	SARS-CoV, WT	0.1	88.0	75-116	219	0.86	0.02	0.0063	0.0002	0.999	**Yes**	158.7	4.9
40S-HCV+0	25	Bat-Hp Zhejiang, WT	0.1	278	234-351	215	0.84	0.01	0.0019	0.0004	0.996	**Yes**	526.3	91.5
40S-HCV(ΔII)+0	25	SARS-CoV-2, WT	0.1	103.0	79-122	214	0.61	0.02	0.0034	0.0001	0.999	**Yes**	294.1	8.4

Supplementary Table 4. Related to Figure 4 and Supplementary Figure 5

Related to Figure 4B

Tethered complex (30 °C)	NSP1 (nM)	Traces (n)	>= 1 Cy3 event	Fraction	+/- 99% C.I.
40S-HCV +0 (rep. 2)	25	1061	875	0.82	0.03
40S-HCV +6	25	1004	812	0.81	0.03
40S-HCV +6, + eIFs	25	1005	885	0.88	0.03
40S-HCV +12	25	1003	877	0.87	0.03
40S-HCV +12, +eIFs	25	1006	661	0.66	0.04
40S-HCV +24	25	1005	520	0.52	0.04
40S-HCV +48	25	1009	308	0.31	0.04
40S-HCV(ΔII)+48	25	593	283	0.48	0.06
biotin-mRNA-40S, +eIFs	25	923	144	0.16	0.03

Related to Figure 4C,D and Supp. Fig. 5G,H

		Association rates and amplitudes from double-exponential fits										Median association time			
Tethered complex (30 °C)	NSP1 (nM)	Amp. 1 (A1)	A1 error	Fast rate (k1, per s)	k1 error	Amp. 2 (A2)	Slow rate (k2, per s)	k2 error	adj. R2	n	Type of fit	Median (s)	95% CI (s)	1/fast rate (1/kon)	Error
40S-HCV +0 (rep. 2)	25	0.40	0.01	0.094	0.006	0.60	0.0068	0.0002	0.998	218	DE	28.2	22.8 to 49.9	10.7	0.6
40S-HCV +6	25	0.25	0.01	0.130	0.01	0.75	0.0054	0.0001	0.998	208	DE	75.7	54.5 to 97.3	7.7	0.7
40S-HCV +6, + eIFs	25	0.51	0.02	0.067	0.005	0.49	0.0082	0.0005	0.997	216	DE	29.25	23.0 to 34.4	15.0	1.0
40S-HCV +12	25	0.18	0.02	0.044	0.007	0.82	0.0066	0.0001	0.999	216	DE	76.6	65.2 to 106.8	23.0	3.0
40S-HCV +12, +eIFs	25	0.60	0.01	0.013	0.0004	NA	NA	NA	0.998	116	SE**	135.8	95.3 to 207.2	77.2	2.3
40S-HCV +24	25	NA	NA	0.002	0.00002	NA	NA	NA	0.995	202	Linear estimate	223.5	193.5 to 248.8	511	4.9
40S-HCV +48	25	NA	NA	0.002	0.00002	NA	NA	NA	0.994	200	Linear estimate	260.5	218.3 to 300.8	520	5.6
40S-HCV(ΔII)+48	25	NA	NA	0.002	0.00003	NA	NA	NA	NA	220	Linear estimate	252.5	228.5 to 283.4	500.0	7.4
biotin-mRNA-40S, +eIFs	25	NA	NA	0.002	0.00004	NA	NA	NA	0.984	128	Linear estimate	253.9	203.75 to 285.0	492	11
biotin-40S, +eIFs, Lack mRNA-Cy5	25	0.36	0.03	0.037	0.004	0.64	0.0053	0.0003	0.9952	250	DE	56.45	49.85 to 81.7	26.7	3
biotin-40S, +eIFs, +mRNA-Cy5	25	NA	NA	0.002	0.00008	NA	NA	NA	0.9812	53	Linear estimate	180.2	124.8 to 234.5	468	17

**restricted to events that ocurred within 200 s to focus on fast rate

DE = double exponential

SE = single exponential

Related to Supp. Fig 5B,G,H

		Lifetime			Estimated lifetimes from the predominate phase yielded by fits to double-exponential functions						
Tethered complex (30 °C)	NSP1 (nM)	Median (s)	95% CI (s)	n	Amplitude	Amp. Error	Rate (k1, per s)	k1 error	adj. R2	Excitation laser power (532 nM)	Limited by dye stability
40S-HCV +0 (rep. 2)	25	38.2	29.4 to 50.5	218	0.86	0.01	0.015	0.0002	0.999	0.6	Yes
40S-HCV +6	25	48.5	38.3 to 57.9	208	0.79	0.03	0.011	0.0009	0.998	0.6	Yes
40S-HCV +6, + eIFs	25	49.6	39.8 to 57.9	216	0.68	0.1	0.0099	0.001	0.997	0.6	Yes
40S-HCV +12	25	36.7	29.6 to 46.5	216	0.91	0.08	0.017	0.0003	0.999	0.6	Yes
40S-HCV +12, +eIFs	25	53.0	41.7 to 65.5	214	0.93	0.01	0.013	0.0001	0.996	0.6	Yes
40S-HCV +24	25	13.8	11 to 15.7	202	0.60	0.04	0.12	0.01	0.997	0.6	No
40S-HCV +48	25	11.9	9.3 to 14.8	200	0.50	0.02	0.25	0.02	0.997	0.6	No
40S-HCV(ΔII)+48	25	45.2	39.5-55.4	220	0.92	0.01	0.014	0.0001	0.998	0.6	Yes
biotin-mRNA-40S, +eIFs	25	16.2	10.9 to 24.6	128	0.59	0.02	0.019	0.002	0.996	0.6	No
biotin-40S, +eIFs, Lack mRNA-Cy5	25	47.3	41.2 to 56.1	250	0.98	0.01	0.017	0.001	0.996	0.6	Yes
biotin-40S, +eIFs, +mRNA-Cy5	25	21.2	12.9 to 51.9	53	0.67	0.02	0.015	0.02	0.995	0.6	No
40S-HCV +0, Low laser power (0.16 μW/μm2	25	94.7	75.7 to 127.3	225	0.73	0.02	0.0043	0.0001	0.998	0.6	Yes

Supplementary Table 5. Related to Figure 5 and Supplementary Figure 7

Related to Supp. Figure 7D

complex (30 °C)	[NSP1] (nM)	Traces (n)	>= 1 Cy5.5 event	Fraction	+/- 99% CI
40S-HCV+0	25	1006	723	0.72	0.04
40S-CrPV+1	25	1004	451	0.45	0.04
80S-CrPV+1	25	385	113	0.29	0.06
80S-CrPV+48	25	875	137	0.16	0.03

Related to Figure 4F

		Association rates and amplitudes from double-exponential fits										Median association time	
complex (30 °C)	NSP1 (nM)	Amp. 1 (A1)	A1 error	Fast rate (k1, per s)	k1 error	Amp. 2 (A2)	Slow rate (k2, per s)	k2 error	adj. R2	n	Type of fit	Median (s)	95% CI (s)
40S-HCV+0	25	0.27	0.01	0.059	0.01	0.73	0.0064	0.0001	0.998	220	DE	63.5	46.2 to 82
40S-CrPV+1	25	0.09	0.01	0.081	0.04	0.91	0.0042	9E-05	0.994	203	DE	153.7	118.6 to 189.2
80S-CrPV+1	25			0.0019	0.00001				0.938	113	*Linear estimate	259.3	235.4 to 285.3
80S-CrPV+6	25			0.002	0.00001				0.959	161	*Linear estimate	264.5	242.9 to 292.7
80S-CrPV+1	25			0.001783	0.00005				0.912	137	*Linear estimate	320.95	288.3 to 353.6

DE = double exponential

Supplementary Table 6. Related to Figure 6 and Supplementary Figure 8

Related to Figure 6A and Supp. Fig. 8M

Tethered RNA (30 °C)	40S:NSP1 (nM:nM)	40S under 200 s	40S-NSP1 under 200s	2nd NSP1 >= 20s	›P1-40S subunit co-associati Fraction	99% CI	NSP1 re-association Rebinding efficiency	99% CI
HCV+0	0	ND	ND	ND	ND	ND	ND	ND
HCV+0	15:30	500	285	227	0.57	0.51-0.63	0.80	0.74-0.86
HCV+48	15:30	500	299	96	0.60	0.54 to 0.66	0.32	0.25-0.39
CrPV+1	0	ND	ND	ND	ND	ND	ND	ND
CrPV+1	15:30	502	315	ND	0.63	0.57-0.69	ND	ND
CrPV+48	0	ND	ND	ND	ND	ND	ND	ND
CrPV+48	15:30	503	309	ND	0.61	0.55-0.67	ND	ND
M+41	15:30	500	9	0	0.02	0.004-0.03	0	0

*ND = not determined

Related to Figure 6C,D and Supp. Fig. 8E,F,I,L

Tethered RNA (30 °C)	40S:NSP (nM:nM)	Parameter	Lifetime Median (s)	95% C.I.	to double-exponential functions Amplitude	Amp. Error	Rate (k1, per s)	k1 error	adj. R2	1/Koff	+/-95% C.I.
HCV+0	15:0	40S subunit	193.8	178.8 to 234.2	1.1	0.01	0.0033	0.00006	0.999	303.0	5.4
		NSP1	ND	ND	ND	ND	ND	ND	ND	ND	ND
		Re-associated NSP1	ND	ND	ND	ND	ND	ND	ND	ND	ND
HCV+0	15:30	40S subunit	217	186 to 251.6	1	0.01	0.0034	0.00005	0.999	294.1	4.3
		NSP1	54.4	38.8 to 74	0.73	0.01	0.0072	0.0002	0.998	138.9	3.8
		Re-associated NSP1	112.2	85.6 to 146.6	0.89	0.01	0.0069	0.00009	0.999	144.9	1.9
HCV+48	15:30	40S subunit	299	241.2 to 360.2	1.2	0.01	0.0018	0.00006	0.998	555.6	17.9
		NSP1	64	52.5 to 81.8	0.73	0.01	0.0067	0.00009	0.999	149.3	2.0
		Re-associated NSP1	88.6	23.3 to 38.4	0.93	0.02	0.0090	0.0002	0.999	111.1	2.4
CrPV+1	15:0	40S subunit	84	66.8 to 97.2	0.73	0.09	0.0062	0.0005	0.998	161.3	12.0
		NSP1	ND	ND	ND	ND	ND	ND	ND	ND	ND
CrPV+1	15:30	40S subunit	106.4	99.6 to 138.4	1	0.01	0.0063	0.0001	0.999	158.7	2.5
		NSP1	40	30.6 to 54	0.7	0.01	0.0091	0.0002	0.999	109.9	2.4
CrPV+48	15:0	40S subunit	136.2	112.8 to 179.2	0.99	0.01	0.0050	0.00009	0.998	200.0	3.5
		NSP1	ND	ND	ND	ND	ND	ND	ND	ND	ND
CrPV+48	15:30	40S subunit	87.7	78.3 to 109.3	0.97	0.01	0.0084	0.0002	0.997	119.0	2.8
		NSP1	29.5	21.9 to 39	0.5	0.04	0.0099	0.0008	0.998	101.0	7.6
M+41	15:0	40S subunit (>5 s)	149.2	121.2-180.3	0.92	0.01	0.0045	0.00008	0.999	222.2	3.9
	15:30	40S subunit (>5 s)	126.2	89.4-163.7	0.68	0.03	0.0038	0.0002	0.997	263.2	13.2
	15:30	40S subunit (with NSP1)	0.4	0.2-0.4	0.81	0.04	4.3	0.9	0.992	0.2	0.04
		NSP1	0.4	0.2-0.4	0.81	0.04	4.3	1	0.99	0.2	0.04

Related to Fig. 6D and Supp. Fig. 8D,F,I,L

Tethered RNA (30 °C)	40S:NSP (nM:nM)	Parameter	Association time (1/k1) (s)	+/- 95% C.I.	Association rates and amplitudes from fits Amp. 1 (A1)	A1 error	Fast rate (k1, per s)	k1 error	Amp. 2 (A2)	Slow rate (k2, per s)	k2 error	adj. R2	n	Type of fit	Median association time Median (s)	95% CI (s)
HCV+0	15:0	NSP1-40S co-association	77	7.7	0.64	0.1	0.013	0.001	0.36	0.0043	0.0008	0.999	213	DE	76.2	58.6 to 93.6
		NSP1 re-association	ND	ND	ND	ND	ND	ND	ND	ND	ND	ND	ND	ND	ND	ND
HCV+0	15:30	NSP1-40S co-association	60.7	3.9	0.68	0.04	0.016	0.001	0.32	0.0030	0.0004	0.997	227	DE	66.8	56.2 to 76.4
		NSP1 re-association	12.7	1.4	0.20	0.01	0.079	0.010	0.80	0.0053	0.0001	0.998	209	DE	93.4	67.2 to 120.2
HCV+48	15:30	NSP1-40S co-association	48.5	3.4	0.63	0.1	0.021	0.002	0.37	0.0045	0.0005	0.997	218	DE	55.8	44.7 to 65.4
		NSP1 re-association	702.7	13.6			0.0014	0.00003					90	*Linear estimate	335.7	253.8 to 406.9
CrPV+1	15:0	NSP1-40S co-association	3.6	0.17	0.68	0.01	0.28	0.01	0.32	0.0059	0.00038	0.996	217	DE	7	5.4 to 8.1
	15:30	NSP1-40S co-association	2.4	0.13	0.74	0.01	0.41	0.02	0.26	0.0083	0.00090	0.995	193	DE	3.6	3 to 4
CrPV+48	15:0	NSP1-40S co-association	2.3	0.18	0.79	0.02	0.43	0.04	0.21	0.014	0.0036	0.987	220	DE	9.8	9.2 to 10.4
	15:30	NSP1-40S co-association	1.9	0.07	0.76	0.01	0.51	0.02	0.24	0.017	0.0015	0.998	174	DE	3.3	2.8 to 3.8
M+41	15:0	40S subunit (>5 s)	166.7	5.4	1.1	0.1	0.0060	0.0002				0.996	214	SE	116	97.6-137.8
	15:30	40S subunit (>5 s)	208.3	3.4	1.1	0.01	0.0048	0.00008				0.999	216	SE	138.9	122.7-159.8
	15:30	NSP1-40S co-association	454.5	6.1			0.0022	0.00003				0.994	108	*Linear estimate	169.6	121-208.2

DE = double exponential
SE = single exponential

MATERIALS AND METHODS (Expanded)

Molecular cloning. See **SI Appendix, Dataset 1** for all relevant sequences.

NSP1. Codon-optimized SARS-CoV-2 (from accession MN997409.1), SARS-CoV (AY508724), MERS-CoV (JX869059), and Bat-Hp-CoV (KF636752) NSP1 and relevant mutants were purchased as geneblocks from IDT and cloned into a vector purchased from the UC Berkeley QB3 MacroLab (vector 1B) using their standard protocol. Synthetic DNA that encoded wild-type, RK124-125AA mutant, KH164-165AA mutant, and ybbR-tagged sequences were purchased from Integrated DNA Technologies (IDT). All sequences were verified by Sanger sequencing. The resulting plasmids encoded NSP1 proteins tagged on the N-terminus with a 6-histidine tag followed by a TEV protease cleavage site and a short NA linker (NH_2-6His–TEV–NA–NSP1-COOH). As noted, a ybbR tag was included either on the N-terminus (ybbR-NSP1) or the C-terminus (NSP1-ybbR).

HCV IRES. A synthetic DNA was purchased from IDT that contained a 5' flanking sequence (pUC19 backbone sequence), a T7 promoter (TAATACGACTCACTATAG), and the HCV IRES (nts 1-344, including the AUG codon). For HCV IRES(ΔdII) constructs, IRES domain II (CCTGTGAGGAACTACTGTCTTCACGCAGAAAGCGTCTAGCCATGGCGTTAGTATGAGTGTCGTGCAGCCTCCAGG) was replaced with sequence (GGACTTCGGTCC) that encodes a stem and tetraloop to promote folding of IRES domain III. In all constructs, downstream of the AUG encoded the rest of domain IV, the HCV core sequence, and an artificial 3’ extension: AGCACGAATCCTAAACCTCAAAGAAAAACCGCCAGAACCATGGAAGAC. DNA templates that encoded HCV+0, +6, +12, +24, and +48 (relative to the ‘G’ of the start codon, 3'-end indicated by underlined nts) were generated via standard PCRs using NEB Phusion polymerase (25 cycles), a common 5’ primer (upstream of the T7 promoter), and specific 3' primers.

CrPV IRES. A plasmid that encodes the IRES of the intergenic region of CrPV with the first codon (Ala) replaced with a Phe codon (TTC) was described previously[1]. The sequence from CCU (bolded) of the IRES to the 3' end was: **CCT**TTCACATTTCAAGATACCGGCGCCATGGAAGACGCCAAAAACATAAAG. DNA templates were generated as for the HCV IRES, with the underlined nts representing the 3'-terminus. We selected +1 as the shortest segment to promote proper folding of the IRES.

GAPDH nanoLuciferase. The reporter was designed to contain a 5' T7 promoter, the nLuc coding sequence (Promega) flanked by the 5' and 3'UTRs from human GAPDH (NCBI GenBank accession: AF261085), a poly(A) tail, and PmeI and SpeI restriction enzyme consensus sites. The reporter construct was purchased from IDT as a plasmid with an pUCIDT backbone and propagated in the DH5α strain of *E. coli*. DNA sequence identity was confirmed by Sanger sequencing. The plasmid was linearized by restriction digest with SpeI (NEB, #R0133) for templated in vitro transcription.

SARS-CoV-2 nanoLuciferase. Viral DNA (NCBI GenBank accession MN997409.1) constructs were designed such that the nLuc coding sequence was flanked by either the full-length 5'UTR or the subgenomic 5' leader sequence and one of two 3´'UTR sequences, and a poly A tail followed by a SpeI consensus sequence. The full-length 5'UTR incuded the first 27 nt of the viral ORF in order to maintain the predicted stem-loop structure within the 5'UTR. The 3'UTR (L) began after the N protein stop codon and the 3'UTR (S) began after ORF10. Synthetic DNAs were purchased as GeneBlocks from IDT (5' leader constructs) or as Gene Parts from GenScript (5'UTR constructs). All synthetic DNAs had a 5'-terminal XmaI consensus sequence and 3'-terminal HindIII consensus sequence. Restriction digest cloning was used to insert viral reporter DNA into a pUC19 vector (NEB, #R10180S, #R3104S, #M2200S).

NSP1 expression, purification, & labeling

All NSP1 expression plasmids were transformed into OneShot BL21(DE3) cells (Invitrogen) and grown overnight at 37 °C on LB agar plates supplemented with 50 µg/mL kanamycin. Liquid cultures of single colonies were grown to $OD_{600} \approx 0.5$ at 37 °C in LB supplemented with kanamycin. Cultures were shifted to 18 °C for 30 minutes, 0.5 mM IPTG was added, and cultures were grown for 16-20 h at 18 °C. Cells were harvested by centrifugation at 5,000 x *g* for 15 min at 4 °C in a Fiberlite F9 rotor (ThermoFisher, cat. # 13456093). Cells were lysed by sonication in lysis buffer (20 mM Tris-HCl pH 8.0, 300 mM NaCl, 10% (v/v) glycerol, 40 mM imidazole, and 5 mM β-mercaptoethanol), and lysates were cleared by centrifugation at 38,000 x *g* for 30 min at 4 °C in a Fiberlite F21 rotor followed by filtration through a 0.22 µm syringe filter. Clarified lysate was loaded onto a Ni-NTA gravity flow column equilibrated in lysis buffer, washed with 20 column volumes (CV) of lysis buffer, 20 CV of wash buffer (20 mM Tris-HCl pH 8.0, 1 M NaCl, 10% (v/v) glycerol, 40 mM imidazole, and 5 mM β-mercaptoethanol), and 10 CV of lysis buffer. Recombinant proteins were eluted with five sequential CV of elution buffer (20 mM Tris-HCl pH 8.0, 300 mM NaCl, 10% (v/v) glycerol, 300 mM imidazole, and 5 mM β-mercaptoethanol). Fractions with recombinant protein were identified by SDS-PAGE analysis. The relevant fractions were dialyzed overnight at 4 °C into ybbR-labeling buffer (50 mM HEPES-KOH pH 7.5, 250 mM NaCl, 10 mM $MgCl_2$, 10% (v/v) glycerol, and 1 mM DTT) or TEV Buffer (20 mM Tris-HCl pH 8.0, 250 mM NaCl, 10% (v/v) glycerol, 10 mM imidazole, and 5 mM β-mercaptoethanol), as appropriate. Fluorescent labeling via the ybbR tag was performed essentially as described[2,3]. Briefly, 14.5 µM ybbR-NSP1 or NSP1-ybbR were incubated at 37 °C for 90 min with 4 µM Sfp synthase enzyme and 20 µM of either Cy3-CoA, Cy5-CoA, or Cy5.5-CoA substrates, respectively. Free dye was removed via purification over 10DG-desalting columns (Bio-Rad, cat.# 7322010) equilibrated in TEV buffer. Dye-labeled and non-labeled NSP1 proteins were incubated with TEV protease at 22 °C for 1.5 hrs followed by 30 min at 37 °C. TEV protease, Sfp synthase, and the cleaved 6His tag were removed via a subtractive Ni-NTA gravity column equilibrated in TEV buffer, with the flow-through collected. NSP1 proteins were subjected to a final purification step using size exclusion chromatography (SEC) on a Superdex 75 column (23 mL) equilibrated in SEC buffer (20 mM HEPES-KOH pH 7.5, 250 mM KOAc, 10% (v/v) glycerol, and 1 mM DTT). Fractions containing NSP1 were concentrated using a 10 kD MWCO Amicon Ultra centrifugal filter, aliquoted, flash frozen on liquid N_2, and stored at -80 °C. Protein concentrations were determined via absorption at 280 nm using a nanodrop for total protein, and at 548 nM or 646 nM for Cy3 or Cy5/5.5 labeled proteins, respectively. For ybbR-NSP1, labeling efficiencies were 50-70%. For NSP1-ybbR, the labeling efficiency was much lower (<20%), and the protein had reduced translation inhibition activity, which is why it was excluded from single-molecule analyses.

nLuc *in vitro* translation assays

HeLa cell-free translation (ThermoFisher, #88884) reactions setup according to manufacturer's protocol were programed with a final mRNA concentration of 200 nM (endpoint) or 80 nM (real-time). For reactions containing NSP1, an equal volume of NSP1 buffer was added to the paired no NSP1 control reaction to account for buffer effects on IVT activity. Prism8 (Graphpad) was used for dose-response analysis ([inhibitor] vs. response with variable slope (four parameters) nonlinear fit) and statistical analysis (one-way ANVA Turkey's multiple comparisons test, unpaired t-test).

Endpoint assays. IVT reactions were incubated at 37°C for 45 min and then immediately transferred to an ice water bath and diluted 1:1 with cold Glo Lysis Buffer (Promega, #E2661). All samples were brought to room temperature and mixed with a 1:1 volume of nGlow solution (Promega, #N1110). Samples (90% of total volume) were loaded into non-adjacent wells of a 384-well plate. Sample luminescence was measured 7 min post nGlow solution addition using a BioTek Neo2 multi-mode plate reader (25°C, 114LUM1537 filter, gain of 135). Luminescence signal was monitored for an additional 30 min at 3 min intervals to verify luminesce signal of all samples decayed at the same rate.

Real-time assays. HeLa IVT reactions were prepared by addition nGlow substrate to the cell-free translation mix with a 1:10 v/v ratio. Before the addition of mRNA and/or NSP1, the IVT reactions were

transferred to non-adjacent wells in a 384-well plate and equilibrated to 30°C in the plate reader. All other reagents were maintained at 30°C and then added to the IVT reactions according to the order-of-addition assay schematic outlined in **Fig. 1D**. The preincubation (30°C, 2 min) was performed in the plate reader. Kinetic monitoring of the samples (36 min, 15 s intervals) was initiated during the equilibration step. Reagent additions and plate transfer times were noted during the experiment and used to post-synchronize t_0 to

mRNA addition. Data was analyzed in MatLab using the approach developed by Vassilenko *et al.*[4]. The raw data was smoothed using Savitzky-Golay filtering (frame length ≤ 5, 2nd degree polynomial) to maintain the shape and magnitude of the raw data. The plateau value of the 1st derivative of the smoothed curve is equal to the synthesis rate[4,5]. Mean synthesis time, which is lag time between mRNA addition and the initial detection of nLuc signal, is the sum of initiation, elongation, and termination time. Synthesis times were extracted from the data by fitting the 2nd derivative of the smoothed data to a Gaussian distribution using the equation

$$f(x) = \ a * e^{\left(-\left(\frac{x-b}{c}\right)^2\right)}$$

where a = amplitude, b = centroid (median), c = variance[4,6]. Statistical analysis of the real-time nLuc activity was done as described above.

Native gel assays

Native composite agarose-acrylamide gels were prepared as described[7]. Briefly, 2.75% acrylamide (37.5:1), 0.5% Nusieve GTG agarose composite gels were prepared in the following buffer: 25 mM Tris-OAc pH 7.5, 4 mM KOAc, 2 mM $Mg(OAc)_2$, 0.5 mM DTT, 2.5% glycerol, 0.1% (v/v) TEMED, and 0.1% (v/v) fresh ammonium persulfate. The gels were cooled at 4 °C for 20 minutes and allowed to further polymerize at room temperature for 90 min. Prior to removal of combs, gels were placed at 4 °C for 15 min. Gels were run in ice-cold running buffer (25 mM Tris-OAc pH 7.5, 4 mM KOAc, and 2 mM $Mg(OAC)_2$) for 30-60 min at 4 °C. All assays were repeated at least three times. For complex formation, the indicated components were incubated in ribosome assay buffer (30 mM HEPES-KOH pH 7.4, 100 mM KOAc, 2 mM $Mg(OAc)_2$) at 37 °C for 15 minutes. Unless noted, NSP1 with a C-terminal ybbR tag conjugated to a Cy5 dye was used in all gel shift experiments, as C-terminally tagged NSP1 from SARS-CoV was functional in cellular assays[8] and Cy5 provides cleaner signal upon image acquisition with a Typhoon imager. For competition experiments, the competitor protein was pre-incubated with ribosomal subunits at 37 °C for 15 minutes, prior to addition of the labeled protein.

Purification of human eIFs

eIF1 and eIF1A. Plasmids for the expression of recombinant human eIF1 and eIF1A were gifted by Christopher S. Fraser (UC Davis). Detailed cloning and recombinant protein expression and purification methods have been described previously[9]. Changes made to the eIF1 and eIF1A purification scheme include use of a HiTrap SP-HP (Cytiva Lifesciences, #17115201) column for the IEX chromatography step and the addition of a SEC step in which fractions eluted from the IEX column containing either eIF1 or eIF1A, as determined by SDS-PAGE analysis, were pooled and passed over a Superdex 75 10/300 (Cytiva, #17-5174-01) column to remove protein oligomers/aggregates and exchange purified factors into a storage buffer (20 mM Hepes, pH 7.5, 250 mM KOAc, 1 mM dithiothreitol (DTT), and 10% glycerol (v/v)). Purified factors were concentrated to ~ 200 μM using a Millipore centrifugal filter and stored at -80°C.

eIF2 and eIF3. Endogenous initiation factor complexes were purified from 400 mL of HeLa post-nuclear extract that was kindly donated to our group by Robert Tijan (UC Berkeley) using previously detailed methods[9,10]. Additional guidance was received from Christopher S. Fraser (UC Davis).

eIF5. The expression and purification of recombinant eIF5 was carried out using a using a modified protocol based on previously established methods[11]. Alterations to the protocol included substitution of the MonoQ column with a HiTrap Q HP column (Cytiva Lifesciences, #29051325) for the anion exchange chromatography step, and adding a SEC step using a Superdex 200 10/300 (Cytiva Lifesciences, 28-9909-44) column to exchange purified eIF5 in to a storage buffer containing 20 mM Hepes, pH 7.5, 250 mM KOAc, 1 mM DTT, and 10% glycerol (v/v).

eIF3j. Human eIF3j was expressed and purified as done for NSP1, with the following changes. Human eIF3j was codon optimized for expression in *E. coli* and cloned with an N-terminal 6xHis tag and TEV protease cleavage site into a pET28b vector at the NcoI site. Protein was expressed by induction with 0.5 mM IPTG overnight at 17 °C. Following cleavage of the 6xHis tag and the subtractive Ni-NTA step, eIF3j was subjected to a final purification step using SEC on a 23 mL Superdex 75 10/300 (Cytiva, #17-5174-01) column equilibrated in storage buffer (20 mM HEPES-KOH pH 7.5, 150 mM KOAc, 10% (v/v) glycerol, and 1 mM DTT). Fractions with purified eIF3j were identified via SDS-PAGE, concentrated, flash frozen, and stored at -80 °C.

$tRNA_i$. Human $tRNA_i$ was transcribed from a DNA template with a 5'-end T7 promoter and hammer head ribozyme[9]. The plasmid was linearized via digestion with BstNI for transcriptional termination at the 3'-CCA of $tRNA_i$. Purified and linearized plasmid was used as the template for in vitro transcription with T7 polymerase at 37 °C for 4 hrs at 16 mM $MgCl_2$, during which the ribozyme self-cleaved (>80% efficiency). Mature $tRNA_i$ was separated from pre-cursor RNA and cleaved ribozyme via 10% acrylamide gel electrophoresis in the presence of 8M urea. After excision of the $tRNA_i$ band, the RNA was extracted 3x at room temperature for 12 hrs with 300 M ammonium acetate, and ethanol precipitated. $tRNA_i$ was resuspended in 10 mM NaCl,10 mM Bis-tris, pH 7.0 and stored at -80°C. To charge $tRNA_i$ with methionine, 100 μL of 60μM human tRNAi transcript was mixed with 618.6 μL ddH2O, and heated at 95 °C for 2 min, then immediately chilled on ice for 5 min. The resulting solution was mixed with 200 μL of 5x charging buffer (200 mM Tris-HCl pH7.5, 50 mM $Mg(OAc)_2$, 5 mM DTT), 20 μL 100 mM ATP, 30 μL 10 mM L-methionine, 2 μL 1 M $Mg(OAc)_2$ and 29.4 μL 34 μM yeast MetRS[12], and incubated at 30 °C for 45 min. The charging reaction was stopped by addition of 100 μL 3M NaOAc pH5.2. The resulting tRNA was purified by phenol-chloroform-isoamyl alcohol (25:24:1, pH 5.2) extraction and ethanol precipitation. The pellet was resuspended with tRNA storage buffer (10 mM NaOAc pH 5.2, 50 mM $Mg(OAc)_2$) and further purified by passing through BioRad P-6 columns that were equilibrated with tRNA storage buffer. The charging efficiency was ≈70% based on acid urea PAGE analyses[13] of the final tRNA product.

Real-time single-molecule assays using ZMWs

ZMW-based imaging. All real-time imaging was conducted using a modified Pacific Biosciences RSII DNA sequencer, which was described previously[14]. Unless otherwise noted, Cy3 dyes were excited using the 532 nm excitation laser at 0.6 $\mu W/\mu m^2$. Cy5 and Cy5.5 dyes were excited with the 642 nm laser at 0.1 $\mu W/\mu m^2$. In nearly all experiments, data were collected at 10 frames per second. The exception was when the 532 nm laser was used at 0.1 or 0.16 $\mu W/\mu m^2$ to extend Cy3 dye lifetimes and provide a better estimation of lifetimes for NSP1-ribosome interactions; in these cases, data were collected at 3 fps to increase signal to noise ratios. ZMW chips were purchased from Pacific Biosciences. Prior to imaging, all ZMW chips were washed with 0.2% Tween-20 and TP50 buffer (50 mM Tris-OAc pH 7.5, 100 mM KCl). Washed chips were coated with neutravidin by a 5 min incubation with 1 μM neutravidin diluted in TP50 buffer supplemented with 1.3 μM of DNA blocking oligos and 0.7 mg mL^{-1} UltraPure BSA. Chips were washed with TP50 and ribosome assay buffer (30 mM HEPES-KOH pH 7.4, 100 mM KOAc, 2 mM $Mg(OAc)_2$). For imaging, the ribosome assay buffer was supplemented with casein (62.5 μg mL^{-1}) and an oxygen scavenging system[15]: 2 mM TSY, 2 mM protocatechuic acid (PCA), and 0.06 U/μL protocatechuate-3,4-dioxygenase (PCD). In all real-time single-

molecule experiments, fluorescently-labeled NSP1 with an N-terminal ybbR tag was used, since it had translation inhibition and 40S-binding activities similar to the wild-type protein.

Tethered eIF1–40S and eIF1–eIF3j–40S complexes (Figure 2). Prior to tethering, 200 nM biotin-40S subunits were incubated in ribosome assay buffer with 6 µM eIF1 at 37 °C for 20 min. For tethering, 1 nM of pre-formed complexes (by biotin-40S) were incubated on the neutravidin-coated ZMW surface for 15 min at room temperature. Non-tethered complexes were washed away with ribosome assay buffer prior to imaging. Upon start of data acquisition and in the presence of 1 µM eIF1, Cy3-NSP1 was added at the indicated concentrations and temperatures. In experiments with eIF3j, 2.5 µM eIF3j was pre-incubated for ~5 min with tethered ribosomal complexes prior to addition of ybbR-NSP1 labeled with Cy3 dye ('Cy3-NSP1'). Temperatures and laser powers were as indicated.

Tethered ribosomal pre-initiation complexes (Figure 2). Complexes were formed by incubating 40 nM biotin-40S subunits in ribosome assay buffer with the indicated eIFs at 37 °C for 15 min. eIF concentrations were: 1 µM for eIF1, eIF1A, & eIF5; and 200 nM for TC-GMPPNP & eIF3. Tethering was conducted as above. As indicated, imaging was conducted in the presence of: 1 µM eIF1, eIF1A, & eIF5; 100 nM TC-GMPPNP; and 50 nM eIF3. Upon start of data acquisition at 30 °C, Cy3-NSP1 was added at 25 nM final concentration.

Tethered 40S–HCV IRES complexes (Figures 3 & 4). The indicated HCV IRES RNAs were diluted to 200 nM in refolding buffer (20 mM cacodylate-NaOH pH 7.0, 100 mM KCl, and 1 mM EDTA), heated to 95 °C for 2 min, and slow cooled to room temperature (~45 min). Once cooled, 4 mM $Mg(OAc)_2$ was added to quench the EDTA, and RNAs were stored on ice until use. To form complexes, 75 nM of 40S-RACK1-Cy5 subunits were incubated in ribosome assay buffer with 20 nM of the indicated IRES RNAs at 37 °C for 20 min. As indicated ('+eIFs'), 1 µM eIF1, 1 µM eIF1A, 1 µM eIF5, 300 nM TC-GMPPNP, and 240 nM eIF3 were included. Tethering was performed as above, except 1 nM of complex was incubated for 5 min at room temperature. Upon start of data acquisition at 30 °C, the indicated Cy3-NSP1 protein was added at 25 nM (final concentration). As indicated (+eIFs), data acquisition was performed in the presence of 1 µM eIF1, 1 µM eIF1A, 1 µM eIF5, 200 nM TC-GMPPNP, and 150 nM eIF3.

Tethered M+41–40S subunit complexes (Figure 4). The biotinylated model mRNA (M+41) and Cy5-labeled model mRNA (M+41-Cy5) were described previously[16]. M+41 mRNA was refolded by incubation in water for 2 min at 95 °C and then flash cooled on ice prior to use. To form complexes with M+41, 40 nM 40S-RACK1-Cy5 subunits were incubated in ribosome assay buffer with 500 nM M+41, 1 µM eIF1, 1 µM eIF1A, 1µM eIF5, and 400 nM TC-GMPPNP at 37 °C for 20 min. Tethering was performed as above, except 1 nM of complex was incubated for 10 min at room temperature. Upon start of data acquisition at 30 °C, Cy3-NSP1 was added at 25 nM final concentration in the presence of 1 µM eIF1, 1 µM eIF1A, 1 µM eIF5, and 200 nM TC-GMPPNP. For M+41-Cy5, conditions were identical except TC-GMPPNP was present at 200 nM and 100 nM during complex formation and imaging, respectively; and, eIF3 was included at 200 nM and 50 nM. eIF3 was included in these experiments to promote complex formation, which was inefficient.

Tethered 40S–CrPV and 80S–CrPV complexes (Figure 5). CrPV IRES RNAs were refolded as with the HCV IRES. To form 40S–CrPV IRES complexes, 40S-uS19-Cy3 subunits at 75 nM were incubated in ribosome assay buffer with 20 nM of the indicated RNA at 37 °C for 15 min. To form 80S–CrPV IRES complexes, 75 nM 40S-uS19-Cy3, 150 nM 60S-uL18-Cy5, and 20 nM RNA were incubated. Tethering was performed as with the 40S–HCV complexes. Upon start of data acquisition at 30 °C, Cy5.5-NSP1 was added at 25 nM final concentration.

Addition of NSP1–40S subunit complexes (Figure 6). HCV IRES, CrPV IRES, and M+41 model mRNAs were refolded as above. To form NSP1–40S subunit complexes for delivery to the tethered IRESs, 550 nM Cy5.5-NSP1 was incubated in ribosome assay buffer with 275 nM 40S-uS19-Cy3 subunits at 37 °C for 15 min.

For delivery to tethered M+41 mRNA, 275 nM 40S-uS19-Cy3 subunits were incubated with 1 µM eIF1, 1 µM eIF1A, 1 µM eIF5, 550 nM TC-GMPPNP, and +/- 550 nM Cy5.5-NSP1 at 37 °C for 15 min. To tether biotinylated mRNAs, 0.16 nM of refolded IRES or > 1µM M+41 mRNA was incubated on the neutravidin-coated ZMW surface for 5 min. Upon start of data acquisition at 30 °C, either 15 nM of 40S-Cy3 subunits alone or 15 nM of Cy5.5-NSP1–40S-Cy3 pre-formed complexes (final concentrations) were added. With M+41, data were collected in the presence of 1 µM eIF1, eIF1A, and eIF5, and 100 nM of TC-GMPPNP. eIF3 was excluded from these experiments as may inhibit association of the pre-initiation complex with the M+41 mRNA[17].

Data analysis. Experimental movies that captured fluorescence intensities over time were processed using custom MATLAB scripts, as described previously[3,14]. Briefly, ZMWs with the desired fluorescence signals were identified by filtering for the signals at appropriate time points. Individual binding events were assigned manually based on the appearance and disappearance of the respective fluorescence signals. Unless noted, only the first NSP1 binding event longer than ~5 s that occurred within the first 500 s of imaging was analyzed. Unless intractable due to complex stability or inhibition, approximately 1,000 molecules were analyzed to determine binding efficiencies and 200 single molecules were analyzed for kinetic analyses, indicated by single-step photobleach events. Association times were defined as the time elapsed from the addition of the labeled component until a burst of fluorescence for that component. The time of addition is controlled by the instrument and varies from experiment to experiment, but typically occurs within the first 10 s of data acquisition. Lifetimes were defined as the duration of the corresponding fluorescence signal.

Kinetic parameters were extracted by fitting observed data to single- or double-exponential functions as described[14]. On some complexes, the presence of a large, slow association phase made it difficult to derive reliable rates, as amplitudes for the association rates are assigned semi-arbitrarily during the fits. When this occurred, comparisons of median association times were used instead, which better reflected the raw data. In the results section, this is indicated by 'median association times', which only pertains to the indicated final concentration of NSP1. Regardless, all derived association rates, median association times, lifetimes, and the number of molecules examined are reported in **SI Appendix, Tables S1-6**. As indicated in the tables, fits to linear functions were used to estimate very slow association rates observed when NSP1 association was inhibited.

Statistical analyses. To calculate errors for NSP1 binding efficiency (*e.g.*, Figure 3E), bootstrap analyses (n = 10,000) were performed to calculate 99% confidence intervals (C.I.) for the observed proportions using R. To calculate errors for median association times and lifetimes, bootstrap analyses (n = 10,000) were performed to calculate 95% C.I. of the observed median using MATLAB. Reported errors for derived rates represent 95% C.I. yielded from fits to linear, single-exponential, or double-exponential functions, as indicated.

Equilibrium single-molecule analyses using total internal reflection fluorescence microscopy (TIRFM)

Our home-built, prism-based TIRFM instrument has been described previously[15,18,19]. All data were collected at room temperature at 10 frames per second with the EM gain set to 650. To form NSP1–40S subunit complexes, 40 nM biotin-40S subunits were incubated in ribosome assay buffer with 500 nM NSP1-ybbR-Cy5 and 6 µM eIF1 at 37 °C for 15 min. Pre-formed complexes were tethered to the surface of a neutravidin-coated quartz slide as with ZMWs above. Buffer conditions during TIRFM imaging were identical to those with ZMWs. During imaging via excitation with the 647 nm laser, 1 µM eIF1 and 4 nM NSP1-Cy5 (C-terminal ybbR tag) were present. To form 80S–IRES complexes, 100 nM of refolded CrPV IRES[1] biotinylated on the 3'-terminus was incubated in ribosome assay buffer with 250 nM 40S-uS19-Cy3 and 750 nM 60S-uL18-Cy5 subunits. The complex was tethered as above at 1 nM. Emission data were collected in both the Cy3 (donor) and Cy5 (acceptor) channels following excitation of the Cy3 donor dye with the 532 nm laser. Co-localized molecules were identified and analyzed using custom MATLAB scripts. FRET events were assigned

manually to a single state, with single molecules indicated by single-step photobleach events of the donor fluorophore. Background fluorescence intensities were corrected and normalized, and the gamma-corrected FRET efficiency distribution was calculated as described[20].

Ribosome purifications and labeling

40S and 60S ribosomal subunits were purified from the indicated cell lines and labeled with biotin or dyes as described[3]. To specifically conjugate biotin on the ribosomal subunit, purified 40S-RACK1-ybbR subunits were incubated with 4-fold molar excess PEG_{11}-biotin (ThermoFisher #21911) conjugated to co-enzyme A (CoA) and 2-fold molar excess Sfp synthase enzyme. To conjugate fluorophores, purified 40S-RACK1-ybbR, 40S-uS19-ybbR, and 60S-uL18-ybbR subunits were incubated with 4-fold molar excess Cy3 or Cy5 dyes conjugated to CoA and 2-fold molar excess of Sfp synthase. All reactions proceeded for 90 min at 37 °C. To remove free biotin, dyes, and/or Sfp synthase, reactions were layered onto a low-salt sucrose cushion (30 mM HEPES-KOH pH 7.5, 100 mM KOAc, 0.5 M sucrose, 5 mM $Mg(OAc)_2$, and 2 mM DTT), and centrifuged at 287,582 x *g* (90,000 rpm) for 1 hr at 4 °C in a TLA100.2 rotor (Beckman, ref. 362046) in 11x34 mm thick-wall polycarbonate ultracentrifuge tubes (Beckman, ref. 343788). Ribosome pellets were washed once and subsequently resuspended with ribosome storage buffer (30 mM HEPES-KOH pH 7.4, 100 mM KOAc, 5 mM $Mg(OAc)_2$, 6% (v/v) sucrose, and 2 mM DTT), aliquoted, flash frozen in liquid N_2, and stored at -80 °C.

RNA in vitro transcriptions

HCV & CrPV IRES RNAs. Purified PCR products were used as the templates for in vitro transcription with the T7 MEGAScript T7 Transcription kit (ThermoFisher #AMB13345). Standard reaction conditions were used, except each nucleotide was present at a final concentration of 4 mM and the reaction was supplemented with 6 mM of 5'-biotin-G-Monophosphate (Trilink, #N-6003). Reactions were incubated at 37 °C for 2 hours. Transcribed RNAs were purified using the GeneJET RNA Purification Kit (ThermoFisher, #K0732).

Reporter mRNAs for IVT assays. Linearized plasmids were used as templates for in vitro transcription with the MessageMAX T7 ARCA-Capped Message Transcription Kit (Cell Script, # CMMA60710). Transcription reactions were setup using standard conditions and then incubated at 37ºC for 30 min. Transcripts were purified using the MEGAclear Transcription Clean-up Kit (ThermoFisher, AM1908). The integrity and homogeneity of the reporter mRNAs were verified via native- and denaturing-PAGE.

CRISPR-Cas9 & Homology-directed repair

Sequences. To generate the 40S-uS19-ybbR and 60S-uL18-ybbR cell lines, the guide sequences CGCAACACTCACCATCTTGC (uS19/RPS15) and AAAAATCATAGAAAATTGCT (uL18/RPL5) were cloned into the pX458 vector backbone using the published approach[21]. To insert the tandem ybbR and flag tags onto the endogenous copies of the genes, single-stranded DNA ultramer repair templates were purchased from Integrated DNA Technologies that contained about 40-60 nts of flanking sequence on either side of the desired insertion. See **SI Appendix, Dataset 1** for all guide oligo, repair template, and PCR screening oligo sequences.

Transfections, sorting, & screening overview. Approximately 24 h post seeding in a well of a 6-well plate, low-confluency (~30%) wild-type HEK293T cells were transiently transfected (Liopfectamine 3000, ThermoFisher) with 1 µg of the relevant pX458 plasmid and 2 µg of ssDNA repair template. Cells were allowed to recover for 48 hrs. Single, eGFP-positive cells were sorted at the Stanford Shared FACS Facility into a well of a 96-well plate that contained 50% conditioned DMEM(high glucose) medium. Individual colonies were allowed to recover until they were visible by eye (approximately 1.5-2 weeks), upon which colonies were transferred to a well of a 24-well plate. Once confluent, colonies were screened via PCR, Sanger sequencing,

and Western blot analyses. Successfully edited cell lines were expanded and ultimately stored as stocks in 5% DMSO-FBS solution.

PCR screening & Sanger sequencing. Genomic DNA was isolated from candidate cell lines using the QuickExtract DNA Extraction Solution (Lucigen, #QE09050) essentially as described, except 10 µL of resuspended cells were added to 50 µL of QuickExtract solution. Following extraction, 1 µL of gDNA was added to a standard 24 µL 2X GoTaq Green PCR (Promega, #M7123), typically ~35 cycles. Sequences for uS19 and uL18 screening oligos are available in **SI Appendix, Table S6**. Following amplification, PCRs were analyzed using 2% agarose gel electrophoresis. PCR products with the desired insertion size were submitted for Sanger sequencing, following their purification.

Western blots. Whole cell lysates were analyzed via 4-20% SDS-PAGE followed by transfer to PVDF membranes. All antibodies were diluted in 5% Blotting-Grade Blocker (BioRad, #1706404) in TBST buffer. The following primary antibodies were used: anti-RPS15/uS19 at 1:500 from Abcam (ab168361); and, anti-RPL5 at 1:500 from GeneTex (GTX101821). Incubations with primary antibodies were for > 12 h at 4 °C. The secondary antibody was GeneTex Rabbit IgG antibody (HRP) (GTX213110-01) and was used at 1:10,000. Blots were imaged via chemiluminescence.

Polysome profiling assays

Polysome profiling assays were performed as described[3] and in duplicate for the indicated cell lines.

Cell lines and growth

HAP1 cells that express RACK1-yybR were described previously[3]. HAP1 cells were grown at 37 °C with 5% CO_2 in Iscove's Modified Dulbecco's medium (IMDM) (Gibco, #sh30228) supplemented with 10% (v/v) fetal bovine serum, 2 mM L-glutamine (Gibco), and 1X penicillin/streptomycin (Gibco). Wild-type HEK293T cells were purchased from ATCC (CRL-3216) and engineered HEK293T cells are described here. All HEK293T cell lines were grown as HAP1 cells except Dulbecco's Modified Eagle's Medium (DMEM) High Glucose medium (Gibco, #11965092) was used instead. Cell lines were not tested for mycobacterium.

Data sharing plan

All plasmids, data, and custom analysis scripts are available upon reasonable request.

Structural models

All structural models were rendered using ChimeraX [22]. The following PDB models were used: 4UG0[23], 5A2Q[24], 6ZLW[25], 6YAL[26], and 6ZMW[27].

SI Appendix, References

1. Petrov, A., Grosely, R., Chen, J., O'Leary, S. E. & Puglisi, J. D. Multiple Parallel Pathways of Translation Initiation on the CrPV IRES. *Mol. Cell* **62**, 92–103 (2016).

2. Yin, J., Lin, A. J., Golan, D. E. & Walsh, C. T. Site-specific protein labeling by Sfp phosphopantetheinyl transferase. *Nat. Protoc.* **1**, 280–285 (2006).

3. Johnson, A. G. *et al.* RACK1 on and off the ribosome. *RNA* rna.071217.119 (2019). doi:10.1261/rna.071217.119

4. Vassilenko, K. S., Alekhina, O. M., Dmitriev, S. E., Shatsky, I. N. & Spirin, A. S. Unidirectional constant rate motion of the ribosomal scanning particle during eukaryotic translation initiation. *Nucleic Acids Res.* **39**, 5555–5567 (2011).

5. Zydowicz-Machtel, P., Swiatkowska, A., Popenda, Ł., Gorska, A. & Ciesiołka, J. Variants of the 5'-terminal region of p53 mRNA influence the ribosomal scanning and translation efficiency. *Sci. Rep.* **8**, 1533 (2018).

6. Du, Z., Alekhina, O. M., Vassilenko, K. S. & Simon, A. E. Concerted action of two 3' cap-independent translation enhancers increases the competitive strength of translated viral genomes. *Nucleic Acids Res.* **45**, 9558–9572 (2017).

7. Johnson, A. G. *et al.* Fluorescently-tagged human eIF3 for single-molecule spectroscopy. *Nucleic Acids Res.* **46**, (2018).

8. Narayanan, K. *et al.* Severe Acute Respiratory Syndrome Coronavirus nsp1 Suppresses Host Gene Expression, Including That of Type I Interferon, in Infected Cells. *J. Virol.* **82**, 4471–4479 (2008).

9. Fraser, C. S., Berry, K. E., Hershey, J. W. B. & Doudna, J. A. eIF3j Is Located in the Decoding Center of the Human 40S Ribosomal Subunit. *Mol. Cell* **26**, 811–819 (2007).

10. Damoc, E. *et al.* Structural characterization of the human eukaryotic initiation factor 3 protein complex by mass spectrometry. *Mol. Cell. Proteomics* **6**, 1135–1146 (2007).

11. Pisarev, A. V, Unbehaun, A., Hellen, C. U. T. & Pestova, T. V. Assembly and analysis of eukaryotic translation initiation complexes. *Methods Enzymol.* **430**, 147–177 (2007).

12. Acker, M. G., Kolitz, S. E., Mitchell, S. F., Nanda, J. S. & Lorsch, J. R. Reconstitution of yeast translation initiation. *Methods Enzymol.* **430**, 111–145 (2007).

13. Köhrer, C. & Rajbhandary, U. L. The many applications of acid urea polyacrylamide gel electrophoresis to studies of tRNAs and aminoacyl-tRNA synthetases. *Methods* **44**, 129–138 (2008).

14. Chen, J. *et al.* High-throughput platform for real-time monitoring of biological processes by multicolor single-molecule fluorescence. *Proc. Natl. Acad. Sci. U. S. A.* **111**, 664–669 (2014).

15. Aitken, C. E., Marshall, R. A. & Puglisi, J. D. An oxygen scavenging system for improvement of dye stability in single-molecule fluorescence experiments. *Biophys. J.* **94**, 1826–1835 (2008).

16. Wang, J. *et al.* eIF5B gates the transition from translation initiation to elongation. *Nature* **573**, 605–608 (2019).

17. Kumar, P., Hellen, C. U. T. & Pestova, T. V. Toward the mechanism of eIF4F-mediated ribosomal attachment to mammalian capped mRNAs. *Genes Dev.* **30**, 1573–1588 (2016).

18. Marshall, R. A., Dorywalska, M. & Puglisi, J. D. Irreversible chemical steps control intersubunit dynamics

during translation. *Proc. Natl. Acad. Sci.* **105**, 15364 LP – 15369 (2008).

19. Blanchard, S. C., Kim, H. D., Gonzalez, R. L. J., Puglisi, J. D. & Chu, S. tRNA dynamics on the ribosome during translation. *Proc. Natl. Acad. Sci. U. S. A.* **101**, 12893–12898 (2004).

20. Larsen, K. P. *et al.* Distinct Conformational States Underlie Pausing during Initiation of HIV-1 Reverse Transcription. *J. Mol. Biol.* **432**, 4499–4522 (2020).

21. Ran, F. A. *et al.* Genome engineering using the CRISPR-Cas9 system. *Nat. Protoc.* **8**, 2281–2308 (2013).

22. Goddard, T. D. *et al.* UCSF ChimeraX: Meeting modern challenges in visualization and analysis. *Protein Sci.* **27**, 14–25 (2018).

23. Khatter, H., Myasnikov, A. G., Natchiar, S. K. & Klaholz, B. P. Structure of the human 80S ribosome. *Nature* **520**, 640–645 (2015).

24. Quade, N., Boehringer, D., Leibundgut, M., Van Den Heuvel, J. & Ban, N. Cryo-EM structure of Hepatitis C virus IRES bound to the human ribosome at 3.9-Å resolution. *Nat. Commun.* **6**, 1–9 (2015).

25. Thoms, M. *et al.* Structural basis for translational shutdown and immune evasion by the Nsp1 protein of SARS-CoV-2. *Science* **8665**, 1–11 (2020).

26. Simonetti, A., Guca, E., Bochler, A., Kuhn, L. & Hashem, Y. Structural Insights into the Mammalian Late-Stage Initiation Complexes. *Cell Rep.* **31**, 107497 (2020).

27. Brito Querido, J. *et al.* Structure of a human 48S translational initiation complex. *Science* **369**, 1220–1227 (2020).
